# Serum RNA Profile Reflects Fluid Status and Atrophic Retinal Changes in Neovascular Age-Related Macular Degeneration

**DOI:** 10.3390/ijms26104852

**Published:** 2025-05-19

**Authors:** Hanna Heloterä, Joanna Kostanek, Mikko Liukkonen, Leea Siintamo, Suvi Linna-Kuosmanen, Cezary Watala, Janusz Blasiak, Kai Kaarniranta

**Affiliations:** 1 Department of Ophthalmology, University of Eastern Finland, 70210 Kuopio, Finland; hanna.helotera@uef.fi (H.H.); mikko.liukkonen@uef.fi (M.L.);; 2 Department of Haemostatic Disorders, Medical University of Lodz, 90-419 Lodz, Poland; joanna.kostanek@stud.umed.lodz.pl (J.K.);; 3 A. I. Virtanen Institute for Molecular Sciences, University of Eastern Finland, 70211 Kuopio, Finland; suvi.linna-kuosmanen@uef.fi; 4 Faculty of Medicine, Collegium Medicum, Mazovian Academy in Plock, 09-402 Plock, Poland; j.blasiak@mazowiecka.edu.pl; 5 Department of Ophthalmology, Kuopio University Hospital, 70210 Kuopio, Finland

**Keywords:** age-related macular degeneration, serum RNA, retina, aging, RNA sequencing, differentially expressed RNAs

## Abstract

The increasing prevalence of age-related macular degeneration (AMD), a disease that can result in the loss of central vision, is an emerging problem worldwide due to aging societies. Growing patient numbers create a challenge for the healthcare system. Understanding the mechanisms of AMD pathogenesis will aid in early, personalized, and efficient intervention, helping to mitigate this issue. Current diagnostic methods rely on optical coherence tomography and angiography imaging, which identify existing damages, but do not provide information on the mechanisms behind them. In the present work, we demonstrate a difference in the serum RNA profile between neovascular AMD (nAMD) patients and controls. Moreover, the RNA profile of nAMD patients corresponded with anatomical changes in the retinal fluid compartments as well as atrophic changes of the retina. We followed two independent ways to control false positive leads, and when these approaches were combined, thioredoxin-related transmembrane protein 4 (TMX4) was observed to be differentially expressed by both approaches. This finding opens a new pathway in AMD studies, which are limited due to restricted access to live human target material and the limited value of animal models of human AMD.

## 1. Introduction

Age-related macular degeneration (AMD) is a progressive retinal disease that affects the macula, the central portion of the retina responsible for color and sharp vision. The etiology of the disease is not fully understood. Still, the development of AMD is believed to be multifactorial involving mechanisms such as RPE dysfunction due to oxidative stress, altered lipid metabolism, low-level inflammation, and complement activation. Clinically, AMD is classified into two forms: dry (80–90%) and neovascular AMD (nAMD; 10–20%), with nAMD progressing rapidly and affecting vision if untreated [1,2,3,4]. Currently, AMD is the leading cause of vision loss in individuals over 55 years, creating significant medical and socioeconomic challenges worldwide. Due to aging populations and the emerging impact of environmental risk factors, the number of affected individuals is projected to rise, causing a burden for ophthalmology clinics [2,3]. Therefore, a more detailed understanding of the mechanisms of AMD pathogenesis is needed for early, personalized, and more efficient medical intervention [5].

Choroidal neovascularization (CNV) and retinal edema are clinical hallmarks of nAMD, which is treated with intravitreal injections of vascular endothelial growth factor (VEGF) inhibitors [2,3,4]. Retinal edema is identified as intra- or subretinal fluid [5]. Typically, early and intermediate stages of AMD do not present clinical symptoms. Late-stage dry or nAMD results in severe vision impairment, affecting visual acuity in activities like reading, face recognition, driving, and watching TV [2]. Recently, classifications and nomenclature for incomplete or complete outer retina atrophy (iORA or cORA) and incomplete or complete retinal pigment epithelium (RPE) and outer retinal atrophy (iRORA or cRORA) were introduced [6]. The degeneration of RPE, photoreceptors, and choriocapillaris is characteristic of AMD pathology [1].

AMD diagnosing methods rely heavily on optical coherence tomography (OCT) and angiography imaging, which identifies damage, but does not provide details at the molecular level [2]. Currently, developments in clinical AMD diagnostics are more focused on imaging, artificial intelligence-based image analysis, the role of imaging markers in the prediction of treatment outcomes, and self-monitoring devices [7,8,9]. Serum biomarkers are not used utilized in the diagnosis of nAMD. Serum as a source for biomarkers is a desirable target since its acquisition requires a far less invasive procedure than samples obtained from the target tissue. More diagnostic biomarkers for nAMD are needed for both research use and to identify patients at high risk for rapid disease progression, a more personalized treatment approach. Emerging evidence points at the serum or blood proteins, lipids, and microRNAs as being associated with macular degeneration [10,11,12,13,14,15]. Although circulating non-coding RNAs (ncRNAs), such as miRNAs, are routinely detected in the blood, far less is known about the significance of circulating mRNAs [14,16,17].

We recently showed that serum mRNA profiles differ between nAMD and control patients and gained initial evidence that serum mRNA may reflect changes in retinal compartments [18]. In this study, we analyzed mRNA, long ncRNA (lncRNA), and small ncRNA (sncRNA) profiles in the serum of nAMD patients and associated them with anatomical changes observed at the retinal level. The study aimed at establishing new, easily accessible, and minimally invasive molecular markers of nAMD to facilitate nAMD research.

## 2. Results

### 2.1. Differentially Expressed Serum RNAs in nAMD vs. Controls

To gain a comprehensive understanding of the circulating RNA profile of nAMD patients, serum RNA samples from 60 nAMD patients and 64 control patients were analyzed. Differentially expressed RNAs were defined as those having at least a 2-fold change and a minimum numerical difference of one in median values alongside statistical significance. RNAs were eligible for disease progression analysis only if they were differentially expressed by these criteria between nAMD and control samples. We found 563 mRNAs, 32 lncRNAs, and two sncRNAs that were downregulated in nAMD samples (Supplementary Table S1). Only five mRNAs and no lncRNAs or sncRNAs were upregulated in nAMD samples based on our criteria (Supplementary Table S1). All the previously mentioned RNAs, later referred to as the initial list, were taken for disease progression analysis.

**Figure 1:**
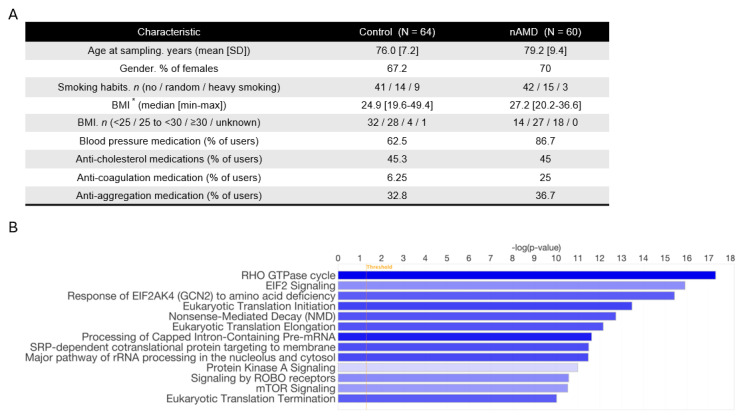

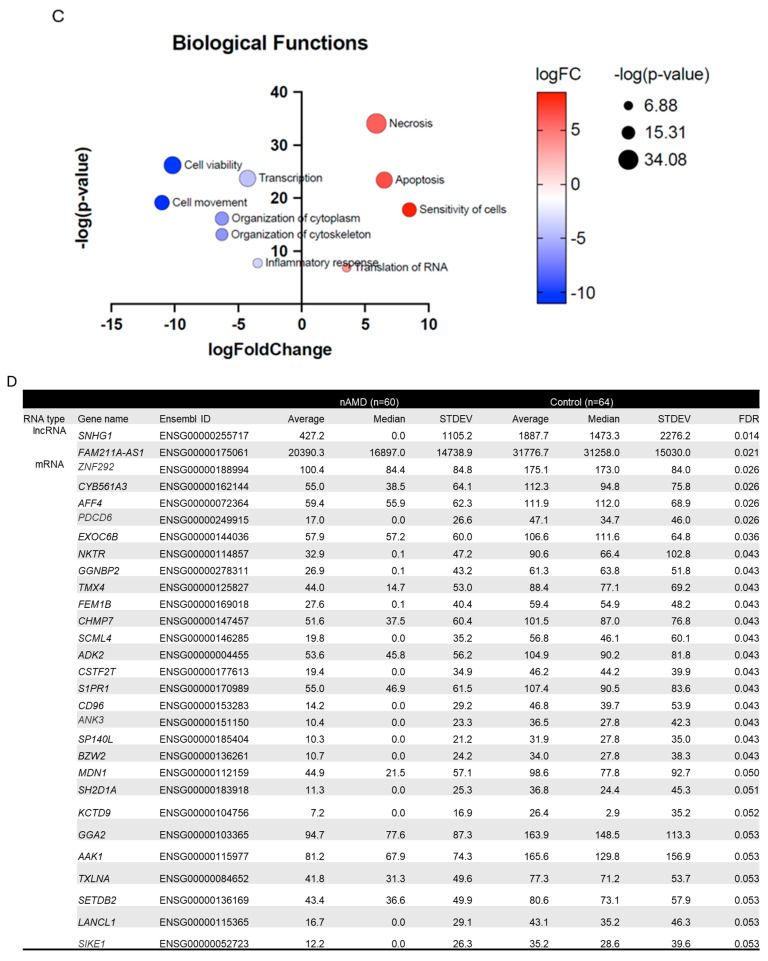
Patient characteristics and mRNAs upregulated in nAMD samples. (**A**) Patient characteristics in the study population. (**B**) Most significantly altered biological functions between nAMD and control samples based on serum RNA analysis. Blue color indicates downregulation in nAMD and the stronger the color, the stronger the association. (**C**) Most significantly altered biological functions in pathway analysis. Blue indicates downregulation and orange color upregulation in nAMD. The color intensity indicates association strength. (**D**) RNAs significant after FDR adjustment. * One value is missing in the nAMD group. Abbreviations: nAMD, neovascular age-related macular degeneration; BMI, body mass index; FDR, false discovery rate.

To evaluate the crosstalk of lncRNAs, sncRNAs, and mRNAs on the pathway level, we performed an integrative analysis of the molecules with ingenuity pathway analysis (Qiagen, Hilden, Germany). Based on the expression patterns of the molecules, the analysis inferred significant downregulation of gene expression and translation of related terms, enriching pathways such as translation initiation and elongation (Figure 1B), thereby explaining the trend of general downregulation of the molecules. Also, pathways important in the regulation of the vasculature, such as the RHO GTPase cycle, were observed to be altered (Figure 1B and  Supplementary Figure S2). Overall, the molecular interactions resulted in increased necrosis and apoptosis and decreased cell viability, movement, and cellular organization (Figure 1C), reflecting degenerative changes observed during AMD.

After false discovery rate adjustment using the Benjamini–Hochberg method, two downregulated lncRNAs and 27 downregulated mRNAs from the initial list remained significant, providing a list of RNAs with a lower likelihood of false leads about differentially expressed RNAs between nAMD and control patients (Figure 1D).

### 2.2. RNA Profile Linked to nAMD Progression

To analyze how the serum RNA profile changes during nAMD progression, the differentially expressed mRNAs in the initial list were further examined based on clinical markers and anti-VEGF treatment (Table 1A). We compared the following conditions: 1. sampling before anti-VEGF treatment vs. sampling during anti-VEGF treatment; 2. patients with only intraretinal fluid (IRF) vs. patients with only subretinal fluid (SRF); 3. patients with iORA vs. patients with cRORA.

Comparing samples taken before the initiation of anti-VEGF treatment (*n* = 23) to samples collected during anti-VEGF treatment (*n* = 34), we discovered that four mRNAs and one lncRNA were downregulated in the samples taken during anti-VEGF treatment (Table 1B). They were also downregulated when nAMD and control samples were compared. None of the sncRNAs were differentially expressed in either the nAMD vs. control or the baseline vs. anti-VEGF samples. Small nucleolar RNA host gene 29 (*SNHG29*), a lncRNA, was downregulated along with Src kinase-associated phosphoprotein 1 (*SKAP1*), phosphoprotein membrane anchor with glycosphingolipid microdomains 1 (*PAG1*), G-rich RNA sequence binding factor 1 (*GRSF1*), and CDK5 regulatory subunit associated protein 2 (*CDK5RAP2*) among the mRNAs. Table 1B shows adjusted values obtained from the ANCOVA analysis. The functions of the downregulated lncRNAs and the proteins of differentially expressed mRNAs are listed in Table 1C [19,20,21,22,23,24].

**Table 1:** Patient characteristics and RNA profile changes due to anti-VEGF treatment. (A) Anatomical characteristics. (B) Altered RNAs at baseline compared to anti-VEGF treatment. Adjusted values from ANCOVA analysis are shown. (C) Highlights of functions of downregulated lncRNA and encoded proteins.

**A**										
**Characteristic**								**nAMD. N = 60**		
Sampling at baseline. n (treatment-naïve)								23		
Sampling after beginning of anti-VEGF treatment. n								37		
Retinal atrophy at sampling. n (iORA/cORA/iRORA/cRORA/RPE tear/unknown)								11/12/12/17/6/2		
Fluid status at sampling. n (IRF/SRF/IRF & SRF/unknown)								18/21/18/3		
**B**										
			**Adjusted means**			**Adjusted means. nAMD**			**Direction of change**	
RNA type	Gene name	Ensembl ID	Control. N = 64	nAMD. N = 59	Adjusted *p*-value. Control vs. nAMD	Baseline	After anti-VEGF	Adjusted *p*-value. Baseline vs. anti-VEGF	in nAMD	after anti-VEGF
lncRNA	*SNHG29*	ENSG00000175061	34,896.0	20,597.8	4.0 × 10^−6^	23,379.0	20,046.7	0.008	Down	Down
mRNA	*SKAP1*	ENSG00000141293	52.3	24.9	0.029	48.0	18.9	3.4 × 10^−6^		
	*PAG1*	ENSG00000076641	81.0	41.8	0.003	57.2	41.1	0.016		
**C**										
**Protein/lncRNA Name**	**Function**							**References**		
*SNHG29*	Inhibits vascular smooth muscle cell calcification. In glioblastoma cells accelerated cell proliferation, migration and EMT process. Enhanced cellular senescence during premature birth.							[19,20,21]		
SKAP1	An immune cell adaptor that regulates T-cell adhesion and optimal cell growth. mRNA downregulated in blood monocytes of nAMD patients.							[25,26]		
PAG1	Hypoxia induced. Interacts with with C-terminal of Src kinase.							[27]		
GRSF1	Regulates cellular senescence during aging. Antagonizes age-related hypercoagulability.							[22,28]		
CDK5RAP2	Involved in cell cycle regulation, cell cycle checkpoint control and DNA repair. Role in development of the eye and retina.							[23,24]		

Abbreviations: nAMD, neovascular age-related macular degeneration; iORA, incomplete outer retinal atrophy; cORA, complete outer retinal atrophy; iRORA, incomplete RPE and outer retinal atrophy; cRORA, complete RPE and outer retinal atrophy; RPE, retinal pigment epithelium; IRF, intraretinal fluid; SRF, subretinal fluid; lncRNA, long non-coding ribonucleic acid; mRNA, messenger ribonucleic acid; *SNHG29*, small nucleolar RNA host gene 29; *SKAP1*, Src kinase-associated phosphoprotein 1; *PAG1*, phosphoprotein membrane anchor with glycosphingolipid microdomains 1; *GRSF1*, G-rich RNA sequence binding factor 1; *CDK5RAP2*, CDK5 regulatory subunit associated protein 2.

**Figure 2:**
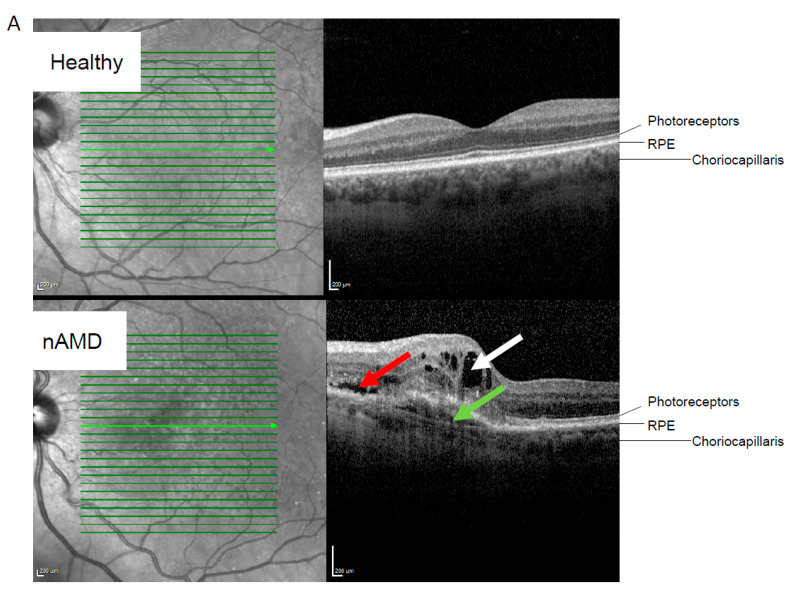

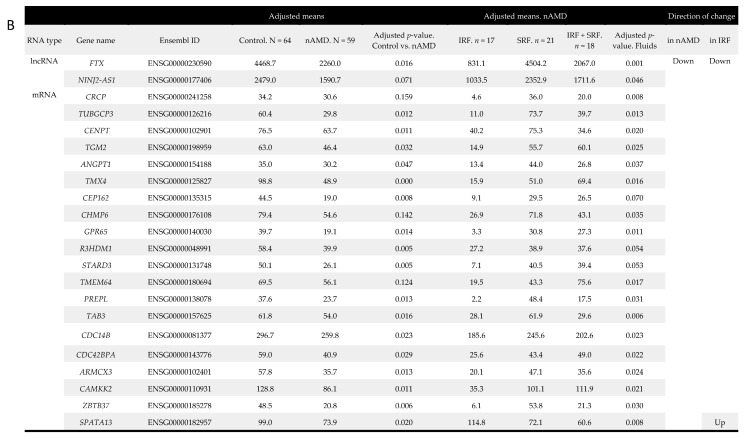

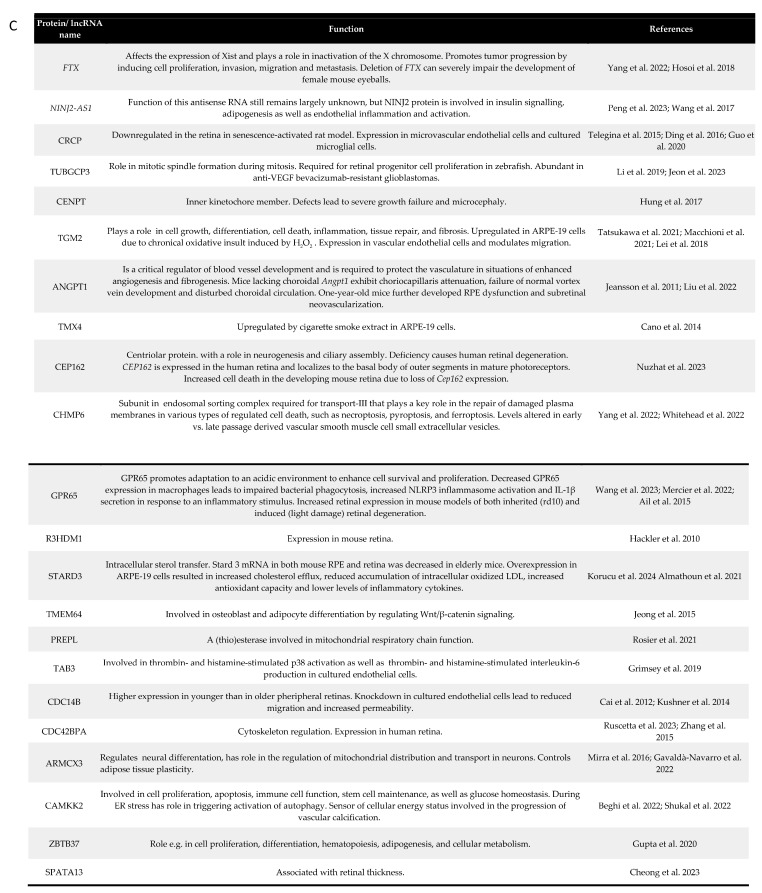
RNA profile differences in the nAMD group based on retinal fluid status. (**A**) Representative optical tomography images from healthy and neovascular AMD (nAMD) cases. The white arrow indicates intraretinal fluid, the red arrow indicates subretinal fluid, and the green arrow indicates subretinal pigment epithelium (sub-RPE) fluid. (**B**) Altered RNAs in SRF vs. IRF analysis. Adjusted values from ANCOVA analysis are shown. (**C**) Highlights of the functions of the altered lncRNAs and encoded proteins by mRNAs. Abbreviations: nAMD, neovascular age-related macular degeneration; IRF, intraretinal fluid; SRF, subretinal fluid; lncRNA, long non-coding ribonucleic acid; mRNA, messenger ribonucleic acid; *FTX*, FTX transcript XIST regulator; *NINJ2-AS1*, NINJ2 antisense RNA 1; *CRCP*, CGRP receptor component; *TUBGCP3*, tubulin gamma complex component 3; *CENPT*, centromere protein T; *TGM2*, transglutaminase 2; *ANGPT1*, angiopoietin 1; *TMX4*, thioredoxin-related transmembrane protein 4; *CEP162*, centrosomal protein 162; *CHMP6*, charged multivesicular body protein 6; *GPR65*, G protein-coupled receptor 65; *R3HDM1*, R3H domain containing 1; *STARD3*, StAR-related lipid transfer domain containing 3; *TMEM64*, transmembrane protein 64; *PREPL*, prolyl endopeptidase-like; *TAB3*, TGF-beta activated kinase 1 (MAP3K7) binding protein 3; *CDC14B*, cell division cycle 14B; *CDC42BPA*, CDC42 binding protein kinase alpha; *ARMCX3*, armadillo repeat containing X-linked 3; *CAMKK2*, calcium/calmodulin-dependent protein kinase kinase 2; *ZBTB37*, zinc finger and BTB domain containing 37; *SPATA13*, spermatogenesis-associated 13 [29,30,31,32,33,34,35,36,37,38,39,40,41,42,43,44,45,46,47,48,49,50,51,52,53,54,55,56,57,58,59,60,61,62,63,64,65,66].

Interestingly, in the IRF only group, nineteen downregulated mRNAs, one upregulated mRNA, and two downregulated lncRNAs were identified when patients with only IRF (*n* = 18) were compared to patients with only SRF (*n* = 21) at the time of sampling (Figure 2). None of the short ncRNAs were differentially expressed in either the nAMD vs. control or IRF only vs. SRF only samples. Figure 2B presents adjusted values obtained from the ANCOVA analysis. The functions of the downregulated lncRNA and encoded proteins of differentially expressed RNAs are listed in Figure 2C [29,30,31,32,33,34,35,36,37,38,39,40,41,42,43,44,45,46,47,48,49,50,51,52,53,54,55,56,57,58,59,60,61,62,63,64,65,66].

**Figure 3:**
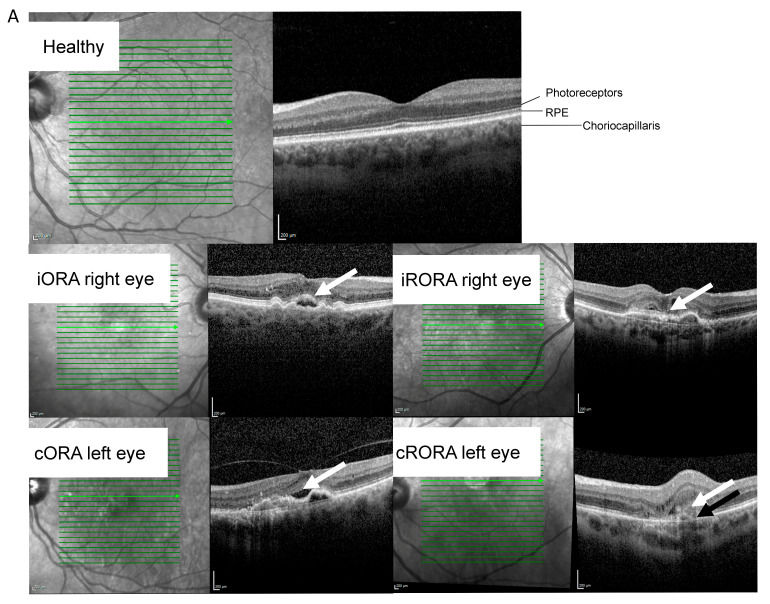

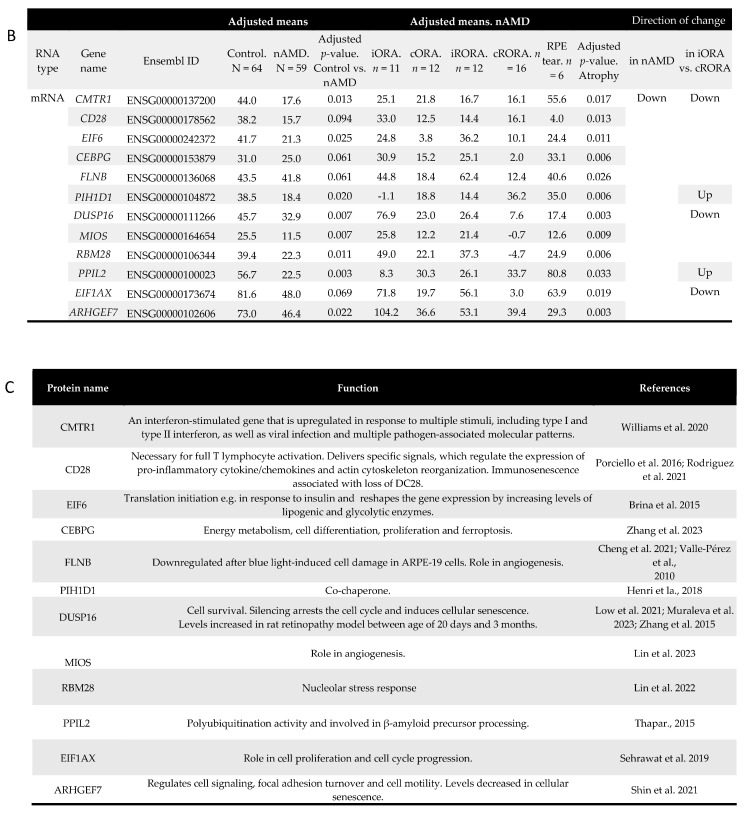
RNA profile differences in the nAMD group during the atrophy analysis. (**A**) Optical coherent tomography images from healthy cases and various levels of atrophy. White arrows indicate outer retina damage, while the black arrow shows changes at the RPE level. Abbreviations: RPE = retinal pigment epithelium, iORA = incomplete outer retinal atrophy, cORA = complete outer retinal atrophy, iRORA = incomplete RPE and outer retinal atrophy, cRORA = complete RPE and outer retinal atrophy. (**B**) Altered RNAs in atrophy analysis. Adjusted values from the ANCOVA analysis are shown. (**C**) Highlights of the functions of the proteins differentially expressed by altered mRNAs. Abbreviations: nAMD, neovascular age-related macular degeneration; iORA, incomplete outer retinal atrophy; cORA, complete outer retinal atrophy cORA; iRORA, incomplete RPE and outer retinal atrophy; cRORA, complete RPE and outer retinal atrophy; *CMTR1*, cap methyltransferase 1; *CD28*, CD28 molecule; *EIF6*, eukaryotic translation initiation factor 6; *CEBPG*, CCAAT enhancer binding protein gamma; *FLNB*, filamin B; *PIH1D1*, PIH1 domain containing 1; *DUSP16*, dual specificity phosphatase 16; *MIOS*, meiosis regulator for oocyte development; *RBM28*, RNA binding motif protein 28; *PPIL2*, peptidylprolyl isomerase-like 2; *EIF1AX*, eukaryotic translation initiation factor 1A X-linked; *ARHGEF7*, Rho guanine nucleotide exchange factor 7 [16,67,68,69,70,71,72,73,74,75,76,77,78,79,80,81].

When patients with iORA (*n* = 11) were compared to patients with cRORA (*n* = 17), ten mRNAs were observed to be downregulated, and two were upregulated in the cRORA group (Figure 3). All atrophy levels, including cRORA and iRORA, were included in the ANCOVA analysis. In the ANCOVA analysis, all identified mRNAs were downregulated in nAMD groups compared to control groups. None of the lncRNAs or short ncRNAs were differentially expressed either in the nAMD vs. control or iORA vs. cRORA samples. Functions of the downregulated lncRNA and proteins encoded by the differentially expressed mRNAs are listed in Figure 3C [16,67,68,69,70,71,72,73,74,75,76,77,78,79,80,81].

### 2.3. Analysis for Covariance

As high variability was observed between the samples, we explored whether gender, body mass index (BMI), smoking status, or the use of blood pressure, anti-cholesterol, anti-coagulation, or anti-aggregation medications could modify observed RNA levels as confounding variables. One sample was excluded from the ANCOVA analyses due to the lack of a BMI value.

When RNAs affected by anti-VEGF treatment were analyzed, all tested variables were identified as confounding factors for some of the tested RNAs (Table 2A and Supplementary Table S2). However, none of the variables affected all tested RNAs. Individual RNAs had their own sets of confounding variables. Similar findings were observed when RNAs affected by atrophy level were analyzed, except that anti-aggregation medication was not a confounding factor for any of the tested RNAs (Table 2B and Supplementary Table S3). This also applied to RNAs identified when samples with IRF and SRF were compared, with the exception that anti-aggregation and anti-cholesterol medications were not identified as confounding factors for any of the tested RNAs (Table 2C and Supplementary Table S4).

For example, BMI was identified as a confounding factor for lncRNA *SNHG29.* Expression levels for *SNHG29* in nAMD (BMI < 25; average = 16,682.3 and SD = 10,476.3; BMI 25 to <30: average = 19,761.8 and SD = 16,283.8; BMI ≥ 30: average = 24,756.8 and SD = 15,058.9) and in controls (BMI < 25: average = 26,524.1 and SD = 13,249.8; BMI 25 to <30: average = 34,689.8 and SD = 13,864.3; BMI ≥ 30: average = 53,407.2 and SD = 14,230.5) showed a rising trend with higher BMIs.

### 2.4. Staining of TUBGCP3 and CHMP6 in the Human Retina, RPE, and Choroid

To evaluate whether protein products of identified mRNAs can be detected from the human eye, TUBGCP3 and CHMP6 were stained from cadaver samples (control patients n = 9, Supplementary Figure S3). TUBGCP3 staining was noted in the retina, photoreceptor outer segments, and in sparsely located RPE cells. The strongest CHMP6 staining was observed in retinal and choroidal blood vessels, photoreceptor outer segments, and random RPE cells. To our knowledge, this is the first report to demonstrate staining of these molecules in the human eye. This analysis provides clues that our approach combining differentially expressed RNAs with information about disease progression status may enrich mRNAs with protein products observed in the posterior segment of the eye.

### 2.5. RNAs Enriched After Both Approaches

To obtain a deeper understanding of serum RNA changes, we followed two approaches to control false leads. In one approach, we used the Benjamini–Hochberg method to adjust differentially expressed RNAs for the false discovery rate. In another approach, we combined our initial list of differentially expressed RNAs with information about disease progression markers. The aim of the latter step was also to evaluate the link between disease progression and the serum RNA profile. When results from both analyses were combined, interestingly, only the RNA that was differentially expressed by both approaches encoded the protein thioredoxin-related transmembrane protein 4 (TMX4).

## 3. Discussion

Unsatisfactory treatment outcomes in nAMD remain one of the greatest challenges in ophthalmology, imposing a significant social and economic burden [1]. To recognize the factors behind the insufficient treatment responses of individual patients, a precise understanding of the processes and biomarkers associated with the disease is needed. We recently showed changes in the serum mRNA profile in nAMD patients, and initial evidence that serum mRNA may reflect changes in retinal compartments was established [18]. In the present study, we analyzed serum RNA profiles in more detail and showed an association of altered signatures of circulating mRNA, ncRNA, and lncRNA with nAMD progression at an anatomical level. Data search criteria differed between this study and our previously published analysis. In this study, we showed distinct serum RNA profiles in patients with nAMD compared to controls. Moreover, we identified mRNAs that were differentially expressed when disease progression was assessed at an anatomical level by comparing different atrophy stages and by comparing samples from patients with either SRF or IRF.

RNA-seq analysis was used to identify serum RNA as it provides the opportunity to identify novel RNA and RNA variants compared to traditional microarray analysis, which analyzes the transcriptome using known RNA probes. Additionally, RNA-seq offers a higher percentage of differentially expressed genes, especially genes with low expression [82]. This is particularly why RNA-seq was chosen for this analysis as serum normally contains only low amounts of RNA. Furthermore, unlike many publications involving RNA-seq analysis, we utilized DNase treatment to eliminate the risk of DNA contamination, which could affect RNA-seq readings [83].

It has been reported that compared to the control group, nAMD patients have different serum and blood miRNA profiles [10,14,15]. Additionally, the response to ranibizumab, an anti-VEGF drug, can be predicted from the miRNA and mRNA profiles of peripheral blood mononuclear cells [84]. In this study, we showed that five hundred sixty three mRNAs, 32 lncRNAs, and two sncRNAs were downregulated, while only five mRNAs were upregulated in AMD samples compared to controls. Interestingly, the mRNAs that were upregulated in nAMD were involved in the inflammatory response, regulation of vasculature, and vesicle transport, highlighting the role of low-level inflammation and disturbed vasculature in the development of nAMD [4,11]. When FDR was adjusted using the Benjamini–Hochberg method, two downregulated lncRNAs and 27 downregulated mRNAs remained significant, providing a list of RNAs that are most likely to yield fewer false leads about differentially expressed between nAMD and control patients. However, to gain a deeper understanding of the serum RNA changes, we also followed another approach to control for false leads. We aimed to perform this step with a larger dataset and accepted a higher number of potential false leads, selecting the initial list without FDR corrections for this process. To minimize the risk that observed changes would reflect conditions other than those caused by AMD, we combined our initial list of differentially expressed RNAs with information about disease progression markers for further analysis. The aim of this step was also to evaluate the link between disease progression and serum RNA profile.

Intravitreal injections with anti-VEGF agents are the standard treatment for nAMD patients. Anti-VEGF agents work by preventing neovascularization, normalizing vessels, and thereby reducing fluid leakage from newly formed or altered capillaries [85]. Since treatment with anti-VEGF agents is considered to slow down disease progression and calm down active disease, we explored if treatment with anti-VEGFs can modify serum mRNA levels for the genes of interest. Intriguingly, the set of genes identified as being associated with nAMD and anti-VEGF response were downregulated both in nAMD and during anti-VEGF treatment. As anti-VEGFs are considered to normalize vasculature, one would expect to see upregulation of genes after anti-VEGF treatment, assuming these genes were downregulated in nAMD. Therefore, we would anticipate a reversal in levels back toward those seen in the control samples. However, it is known that anti-VEGF treatment typically slows disease progression, and thus the observed changes may also result from this inevitable disease progression. One potential source of bias is when intravitreally administrated anti-VEGFs are eliminated from the eye through serum [86]. Although serum concentrations of anti-VEGFs after intravitreal injection are lower than vitreous concentrations, we cannot exclude the possibility that the observed changes were due to low circulating levels of anti-VEGF drugs [87,88].

When comparing fluid compartments in the retina, IRF is considered more detrimental for vision than SRF and is linked to faster AMD progression and increased atrophy [5,89]. Intriguingly, we identified a set of mRNAs and lncRNAs that were expressed differentially when comparing IRF only and SRF only samples. These 22 RNAs, listed in Figure 1, are involved in the maintenance of vasculature and in the development and maintenance of the retina.

The early phase of retinal atrophy characterized by only slight thinning of the outer nuclear layer is represented by iORA [6]. In iORA, a discontinuous loss of the ellipsoid zone is observed, and the interdigitation zone is not visible. In contrast, cRORA manifests at the later stage of atrophy. During cRORA, absence of the RPE along with associated choroidal hypertransmission can be seen together with the findings of cORA [6]. Thus, as iORA and cRORA represent different stages in atrophy development, we compared samples with iORA and cRORA. Interestingly, differences in atrophy levels were associated with altered mRNA expression. Twelve differentially regulated mRNAs, listed in Figure 2, showed functions in a variety of cellular processes, including cell cycle, senescence, and angiogenesis [4,88,90].

Finding that RNA levels differ between the SRF and the IRF groups as well as between different atrophy levels is especially intriguing as they reflect measurable clinical changes in the eye [5]. The fact that serum marker profiles between these comparisons do not overlap reflects most probably the reality that compared changes may exist independently from each other, emphasizing the complexity of nAMD. However, it must be kept in mind that all RNAs identified in fluids and atrophy analyses were already identified to be differentially expressed between control and nAMD patients, which reduces the risk that changes without links to nAMD would be detected. The origin of these RNAs is still unclear, but this finding opens up a new question about whether the identified RNAs originate from the eye or from distal organs. To evaluate if protein products of identified mRNAs can be detected from the human eye, TUBGCP3 and CHMP6 were stained from cadaver samples. Intriguingly, both were identified from multiple compartments of the eye, including photoreceptor outer segments, retinal pigment epithelial cells, and the vasculature of the choroid. According to our understanding, this is the first report to show staining of these molecules in the human eye. This analysis does not answer the question about the origin of identified RNAs but provides clues that our analysis enriches mRNAs with protein products observed in the posterior segment of the eye.

In addition to local detrimental effects, nAMD is known to have systemic components [11,91,92]. For example increasing evidence suggests that systemic risk factors such as smoking, hypertension, lupus erythematosus, Sjogren’s syndrome, giant cell arteritis, Crohn’s disease, diabetes, and obesity are also associated with AMD [5,93,94,95,96,97]. Moreover, high systemic levels of IL-6 are shown to be associated with late AMD, and low serum levels of TNF-α have been described to be linked with increased visual acuity after anti-VEGF therapy for nAMD [98,99]. In addition, we recently reported that patients on anti-coagulation medication receive an nAMD diagnosis later than their comparators, and previously the anti-coagulant dabigatran has been suggested to reduce the risk for new nAMD [5,100]. Thus, increasing evidence suggests that systemic components can also modulate the progression of AMD, and we consider that systemic serum biomarkers should also be evaluated in the future when estimating AMD progression.

Serum as a source of RNA contains only low amounts of RNA, and due to the circulating nature of fluid, it is exposed to a variety of factors that may influence serum transcriptome. As expected, high variance between the samples was observed during analysis. Therefore, it is important to identify confounding factors that can explain at least a part of the variance seen in the individual RNA expression levels between samples. We observed that gender, BMI, and smoking as well as the use of blood pressure, anti-cholesterol, anti-coagulation, or anti-aggregation medications could be confounding factors that should be considered in the analysis of serum RNA data from nAMD patients. For example, BMI was identified as a confounding factor for lncRNA *SNHG29*, whose expression levels were higher in the control samples than in the nAMD samples. In both groups, the higher the BMI, the higher the SNHG29 levels detected. In fact, *SNHG29* expression levels in low BMI controls were comparable to the *SNHG29* expression levels of high BMI nAMD patients (*p* = 0.681). Thus, when comparing *SNHG29* levels from serum samples of nAMD and control patients, only patients from the same BMI group should be compared to reduce variance in expression levels.

In our study, we followed two approaches to limit the number of false leads for future analyses. When results from both analyses were combined, interestingly only RNA that was differentially expressed by both approaches encodes the protein thioredoxin-related transmembrane protein 4 (TMX4). While protein staining for TUBGCP3 and CHMP6 showed staining in the posterior eye, individual approaches may also contain RNAs, which should be examined in more detail in the future. However, if we are willing to start with the fewest false leads for future studies, we would begin with TMX4 as it emerged from both approaches. Notably, the biological functions of TMX4 have not been well established, but it has been previously linked to oxidative damage in cultured RPE cells and has been shown to act as a reductase in vitro [44,101]. Moreover, TMX4 has been shown to interact with calnexin, a protein whose impairment leads to retinal degeneration [101,102]. The role of TMX4 as a reductase and changes in expression during oxidative damage are intriguing as oxidative stress-induced damage is considered to play a key role in AMD development [103].

This study suggests that the expression levels of specific RNAs could indicate altered pathological conditions and particular elements in the disease progression of patients with nAMD. We believe that our findings open new pathways for AMD research and may contribute to novel diagnostic and therapeutic strategies in nAMD.

## 4. Materials and Methods

### 4.1. Patient Blood Sample and Data Collection

The study was approved by the Ethics Committee of the Kuopio University Hospital (approval number 42/2014). To protect personal data, patient information was collected and stored in accordance with the European Union’s Regulation 2016/679. Informed consent was obtained from all subjects involved in the study, and patients provided signed approval for blood sample collection. The study was conducted in compliance with the Declaration of Helsinki.

Blood samples from 60 nAMD patients and 64 controls were obtained at the Kuopio University hospital. Serum samples were collected between 2015 and 2019. Control patients were those undergoing cataract surgery who showed no signs of retinal degeneration. Patient characteristics are displayed in Table 1A. For serum extraction, ten milliliters of blood were collected to BD Vacutainer^®^ Clot Activator Tubes (Becton Dickinson, Franklin Lakes, NJ, USA), gently inverted ten times and allowed to sit for 30–60 min at room temperature. The serum was separated after centrifugation (3200× *g* at 20 °C for 15 min) and transferred into two smaller Mekamini tubes (Mekalasi, Helsinki, Finland). The samples were stored at −70 °C until sequencing. Data on retinal properties were gathered for nAMD patients only.

### 4.2. RNA Sequencing

Serum samples were processed and sequenced at the Institute for Molecular Medicine Finland (FIMM) Genomics NGS Sequencing unit at the University of Helsinki, supported by the Helsinki Institute of Life Science and Biocenter Finland. RNA extraction was conducted at the HiPREP Core at the FIMM and sequenced at the FIMM Genomics NGS Sequencing unit at the University of Helsinki, backed by the Helsinki Institute of Life Science (HiLIFE) and Biocenter Finland. Total RNA was extracted (serum volume 1 mL) using the Maxwell^®^ RSC 48 Instrument (Madison, WI, USA) and Maxwell RSC miRNA Plasma and Serum Kit (Promega, Madison, WI, USA) following the manufacturer’s instructions. The protocol included a DNase treatment. RNA was eluted in 50 µL of RNase-free water (35–40 µL recovered) and sample quality and quantity were determined using Agilent Bioanalyzer 2100 with the RNA 6000 Pico Kit (Agilent Technologies, Santa Clara, CA, USA). The obtained RNA quality and yield were deemed good for a serum extraction (Supplementary Figure S1).

Library preparation from 800 ng of total RNA was performed according to the Illumina Stranded Total RNA with Ribo-Zero Plus Reference Guide (Illumina, San Diego, CA, USA). Library quality checks were performed using the LabChip GX Touch HT High Sensitivity assay (PerkinElmer, Shelton, CT, USA). The libraries were quantified for sequencing using the KAPA Library Quantification Kit (KAPA Biosystems, Wilmington, MA, USA). Sequencing was carried out with Illumina NovaSeq6000 system using S4 flow cell with lane divider (Illumina, San Diego, CA, USA). The data yield from sequencing was satisfactory within sample-type constraints and the read length for the paired-end run was 2 × 151 bp. The RNA-seq datasets were quality-checked and pre-analyzed according to FIMM-RNAseq 2.0.7 workflow [104]. The data discussed in this publication have been deposited in NCBI’s Gene Expression Omnibus (GEO) and are accessible through GEO Series accession number GSE273435 https://www.ncbi.nlm.nih.gov/geo/query/acc.cgi?acc=GSE273435 (accessed on 29 April 2025) [105]. Data validation to determine if RNA-seq data correlates with RT-PCR analysis is performed and described previously by Liukkonen et al. [18].

### 4.3. Data Processing and Statistical Analysis

The acquired count data were filtered to separate protein-coding and non-coding RNAs using data sourced from Ensembl BiomaRt, which were then filtered in R Statistical Software 4.1.3 with DESeq2 1.34.0 to include a minimum of 10 reads in at least 7 patients [106,107,108].

Differentially expressed genes were selected by identifying genes with at least a 2-fold change (downregulated if fold change in median ≤0.5 and upregulated if foldchange in median ≥2) and at least a numerical difference of 1 in median values and statistical significance. RNAs were eligible for disease progression analysis only if they were differentially expressed between nAMD and control samples.

Calculations were performed using SPSS software 27.0.1.0 and Excel 2016 utilizing library size normalized counts per million values acquired with edgeR [109]. Numerical data were expressed as medians  ±  standard deviations. A Student’s *t*-test and a Mann–Whitney *U* test were used to compare the numerical variables. A false discovery rate (FDR) adjustment was made using the Benjamini–Hochberg method. Integrative analysis of the lncRNAs, sncRNAs, and mRNAs was performed with Ingenuity Pathway Analysis (Qiagen, Hilden, Germany).

Given that high variance variability was observed between the samples, data were further checked by the bootstrap-boosted analysis of covariance (ANCOVA) adjusted for selected confounders, including gender, body mass index (BMI), and smoking status and for the use of blood pressure medication, anti-cholesterol medication, anti-coagulation medication, and anti-aggregation medication. Additionally, univariate significance tests were performed. One sample was excluded from the ANCOVA analyses due to a missing BMI value.

### 4.4. Staining of Cadaver Samples

Paraffin-fixed human cadaver samples were stained with CHMP6 (1:100, ab235050, Abcam, Cambridge, UK) and TUBGCP3 (1:100, Sigma Aldrich, St. Louis, MO, USA, HPA043913). Before incubation with the primary antibody, sections were deparaffinized with xylene (3 × 10 min) and rehydrated through an ethanol series (Abs EtOH 2 × 5 min, 94% EtOH 2 × 5 min, 70% EtOH 5 min, distilled H_2_O 5 min). TUBGCP3 sections were pretreated with TRIS buffer (5 min, 90 °C) and washed with TBS (pH 7.4, 2 × 2min). Endogenous peroxidase and alkaline phosphatase were blocked with BLOXALL (Vector laboratories, Burlingame, CA, USA, SP-6000-100, 10 min). Sections were next washed with TBS (5 min) and blocked with 2.5% horse serum (20 min). Primary antibodies were incubated overnight at +4 °C. On the next day, sections were further washed for 5 min with TBS and incubated with the secondary antibody (ImmPRESS polymer kit AP Horse anti-rabbit, MP-5401, Vector Laboratories, Burlingame, CA, USA, 30 min). Next, sections were washed 2 × 5 min with TBS. Signal development was performed using the Vector^®^ Red Substrate Kit, Burlingame, CA, USA, alkaline phosphatase (SK-5100), followed by washing with TBS (5 min), 2× dips in distilled H_2_O, a dip in hematoxylin, and a 3× wash with distilled H_2_O. Sections were placed in TBS before mounting, which was carried out with Aquamount H-5501.

Imaging of the samples was performed at the Biomedicum Imaging Unit, University of Helsinki, with the support of the Helsinki Institute of Life Science (HiLIFE, Helsinki, Finland) and Biocenter (Helsinki, Finland). Images were acquired using a Zeiss (Oberkochen, Germany) Axio Imager microscope with 40× magnification. The microscope contains a Zeiss AxioCam 105 color camera for transmitted light imaging and uses Zeiss Zen 2 software (Oberkochen, Germany).

## Figures and Tables

**Table 2:** Summary of confounding factors for RNAs influenced by anti-VEGF treatment (A), RNAs affected by atrophy level (B), and for RNAs identified when samples with IRF and SRF were compared (C).

A			B			C		
**Confounding Variable**	**Number of RNAs Affected**		**Confounding Variable**	**Number of RNAs Affected**		**Confounding Variable**	**Number of RNAs Affected**	
	Control vs. nAMD	Baseline vs. anti-VEGF		Control vs. nAMD	Atrophy status		Control vs. nAMD	SRF vs. IRF
Gender	0/5	1/5	Gender	1/22	3/22	Gender	1/12	3/12
BMI	2/5	1/5	BMI	2/22	3/22	BMI	1/12	2/12
Smoking	0/5	1/5	Smoking	1/22	2/22	Smoking	0/12	4/12
Blood pressure	1/5	1/5	Blood pressure	1/22	1/22	Blood pressure	1/12	2/12
Anti-cholesterol	0/5	3/5	Anti-cholesterol	1/22	1/22	Anti-cholesterol	3/12	0/12
Anti-coagulants	2/5	3/5	Anti-coagulants	1/22	1/22	Anti-coagulants	0/12	1/12
Anti-aggregation	0/5	2/5	Anti-aggregation	0/22	0/22	Anti-aggregation	2/12	1/12

Abbreviations: nAMD, neovascular age-related macular degeneration; IRF, intraretinal fluid; SRF, subretinal fluid.

## Data Availability

The data discussed in this publication have been deposited in NCBI’s Gene Expression Omnibus (GEO) and are accessible through GEO Series accession number GSE273435 https://www.ncbi.nlm.nih.gov/geo/query/acc.cgi?acc=GSE273435 (accessed on 29 April 2025).

## Supplementary Materials

The following supporting information can be downloaded at: https://www.mdpi.com/article/10.3390/ijms26104852/s1.
