# Supplementary material for: Serum RNA Profile Reflects Fluid Status and Atrophic Retinal Changes in Neovascular Age-Related Macular Degeneration
Source: Int J Mol Sci. 2025 May 19;26(10):4852. doi: 10.3390/ijms26104852 (PMC12112293; doi:10.3390/ijms26104852)
Supplement: Supplementary file 1 [file ijms-26-04852-s001.zip › ijms-3548661-supplementary.pdf]

Supplemental tables S1-4 and supplemental figures S1-3.

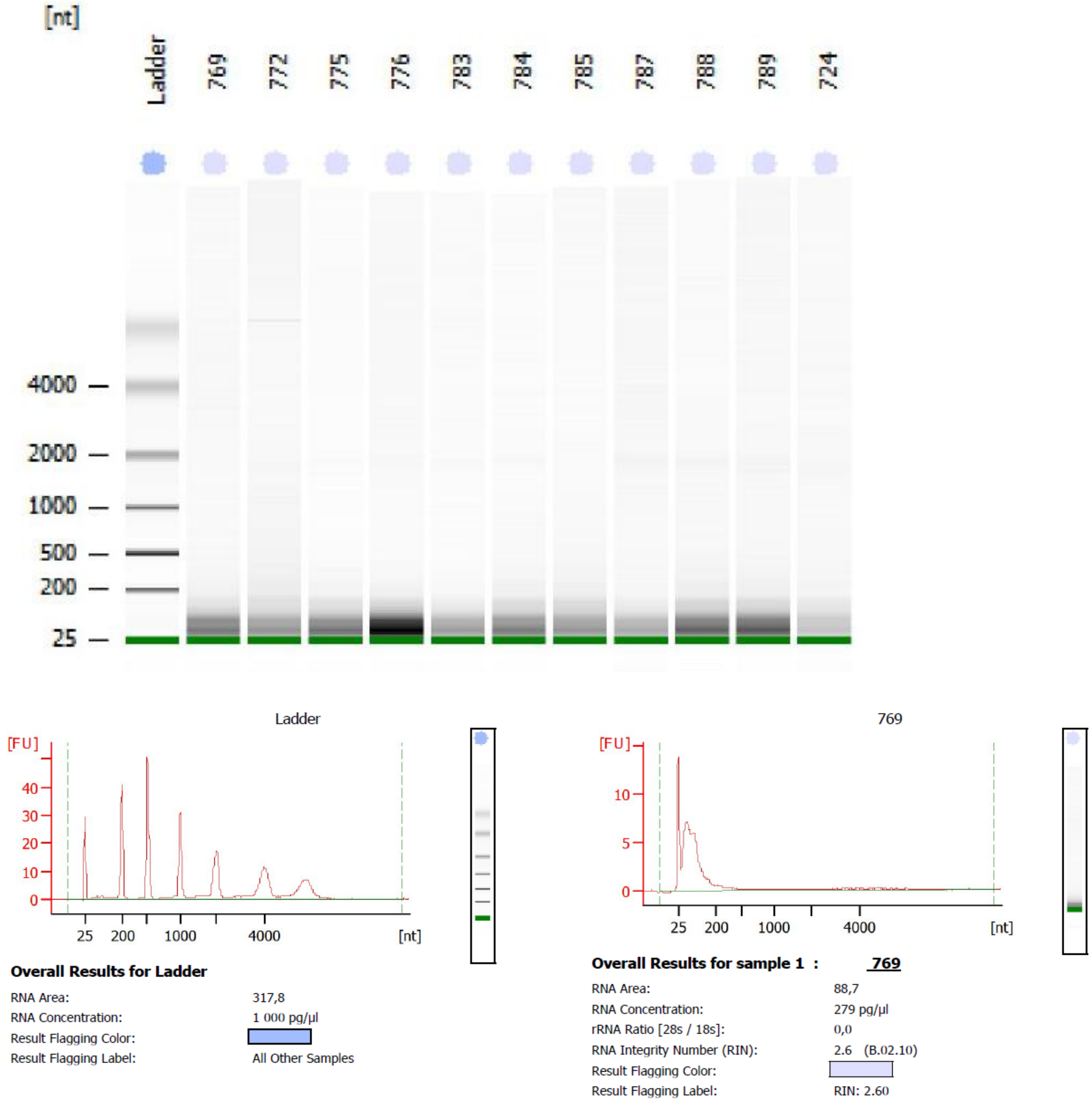

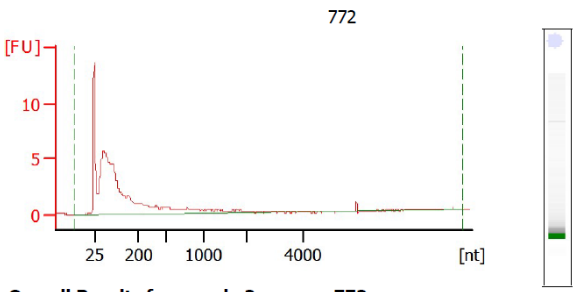

Overall Results for sample 2 : <u>**772**</u>

RNA Area:	76,4
RNA Concentration:	240 pg/µl
rRNA Ratio [28s / 18s]:	0,0
RNA Integrity Number (RIN):	2.5 (B.02.10)
Result Flagging Color:	
Result Flagging Label:	RIN: 2.50

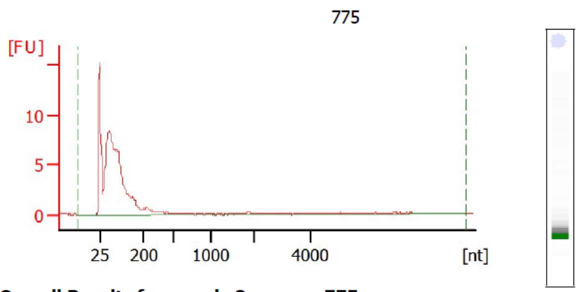

Overall Results for sample 3 : <u>**775**</u>

RNA Area:	92,1
RNA Concentration:	290 pg/µl
rRNA Ratio [28s / 18s]:	0,0
RNA Integrity Number (RIN):	2.6 (B.02.10)
Result Flagging Color:	
Result Flagging Label:	RIN: 2.60

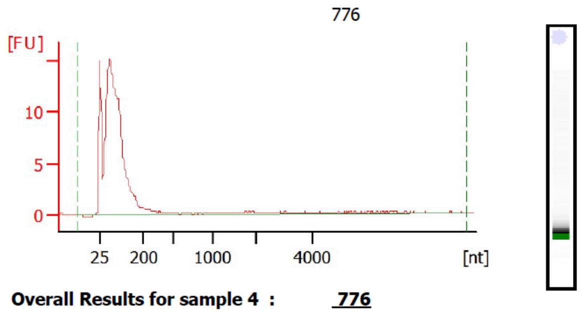

Overall Results for sample 4 : <u>**776**</u>

RNA Area:	152,4
RNA Concentration:	479 pg/µl
rRNA Ratio [28s / 18s]:	0,0
RNA Integrity Number (RIN):	2.6 (B.02.10)
Result Flagging Color:	
Result Flagging Label:	RIN: 2.60

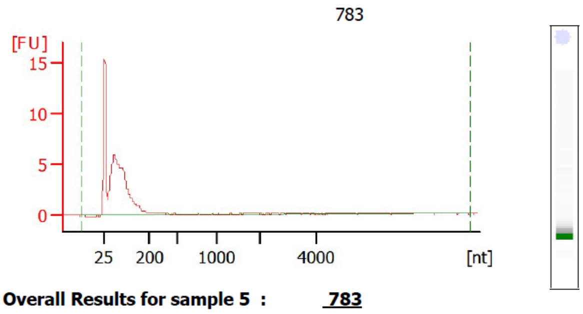

Overall Results for sample 5 : <u>**783**</u>

RNA Area:	63,4
RNA Concentration:	200 pg/µl
rRNA Ratio [28s / 18s]:	0,0
RNA Integrity Number (RIN):	2.5 (B.02.10)
Result Flagging Color:	
Result Flagging Label:	RIN: 2.50

Supplemental Figure S1. Examples of RNA quality and yield. RNA was carefully extracted from the serum after removing the cells. High-quality RNA obtained from cell-free serum differs from that extracted from cells. Since cells are removed from the serum before RNA extraction, RIN is not a reliable measurement for RNA quality in serum samples. RNA quality and yield were considered good in the case of serum extraction.

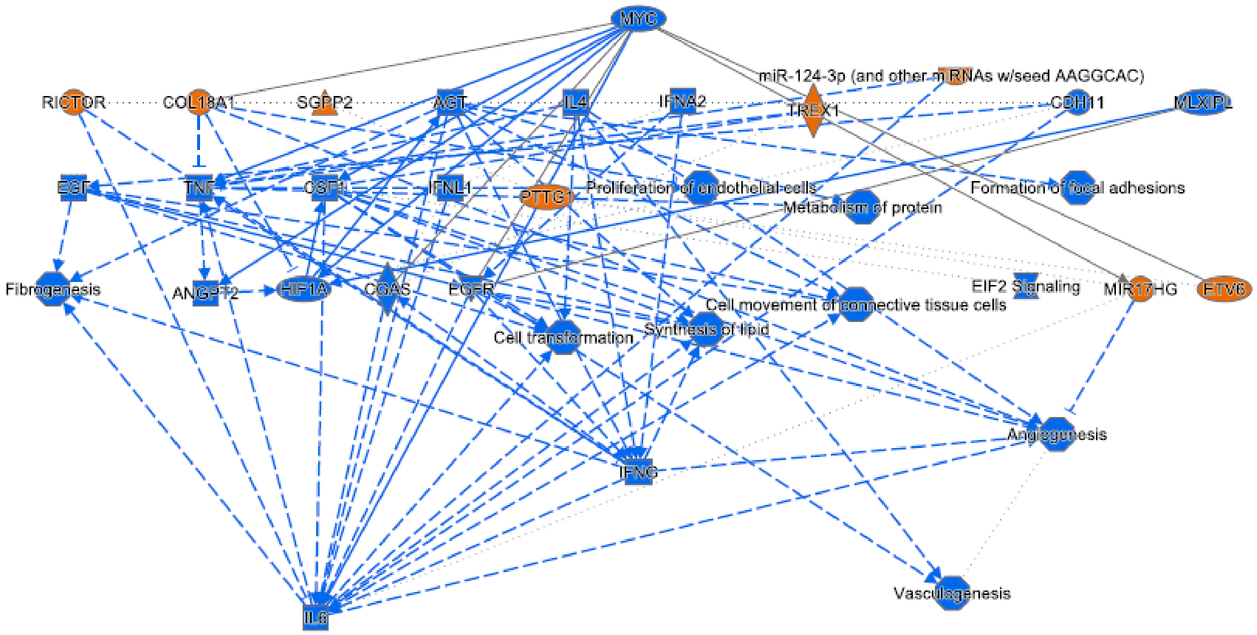

Supplemental Figure S2. Machine learning analysis about affected pathways created by with Ingenuity Pathway Analysis (Qiagen). Orange color indicates activation and blue color inhibition. Direct lines are direct connections, and dashed lines are indirect connection, which may include other molecules as a connector. Dotted line indicates predicted connection.

Supplemental Table S1. RNAs upregulated (A) and downregulated (B) in nAMD (control vs. nAMD). From downregulated only the 200 mRNAs with the greatest difference between the medians of control and nAMD are shown. * Fold change could not be calculated as the other median is zero. Abbreviations: nAMD, neovascular age-related macular degeneration; lncRNA, long non-coding ribonucleic acid; mRNA, messenger ribonucleic acid; ncRNA, non-coding RNA. *OSM*, oncostatin M; *RILP*, Rab interacting lysosomal protein; *GZMH*, granzyme H; *G0S2*, G0/G1 switch 2; *MAP3K8*, mitogen-activated protein kinase 8; *DOC2B*, double C2 domain beta

A

			nAMD (N = 60)			Control (N=64)			nAMD vs. Control			
RNA type	Gene name	Ensembl ID	Average	Median	STDEV	Average	Median	STDEV	Fold change in medians	Difference in medians	*p* (ttest)	*p* (utest. asymp.)
mRNA	*OSM*	ENSG00000099985	159.9	106.5	171.3	88.1	49.3	102.6	2.2	57.2	0.006	0.007
	RILP	ENSG00000167705	49.3	39.2	72.1	27.7	0.1	46.9	298.1	39.1	0.052	0.029
	GZMH	ENSG00000100450	44.2	29.9	53.8	23.1	0.0	38.8	2724.4	29.9	0.015	0.045
	G0S2	ENSG00000123689	189.9	142.7	198.0	121.9	58.2	147.8	2.4	84.4	0.033	0.046

	MAP3K8	ENSG00000107968	63.6	52.3	63.7	36.0	25.7	39.6	2.0	26.6	0.005	0.047
	DOC2B	ENSG00000272636	39.1	2.6	53.3	19.4	0.0	37.7	*	2.6	0.020	0.033

B

		nAMD (N = 60)			Control (N= 64)			nAMD vs. Control			
RNA type	Ensembl ID	Average	Median	STDEV	Average	Median	STDEV	Fold change in medians	Difference in medians	*p* (ttest)	*p* (utest, asymp.)
lncRNA	ENSG00000251562	126104.6	66745.0	136837.2	281837.6	156817.1	359058.3	0.426	90072.1	0.002	0.000
	ENSG00000175061	20390.3	16897.0	14738.9	31776.7	31258.0	15030.0	0.541	14360.9	0.000	0.000
	ENSG00000251600	6570.9	4423.0	7121.2	11738.3	10053.7	9193.7	0.440	5630.7	0.001	0.001
	ENSG00000281649	3071.7	615.3	4361.8	4850.8	3592.9	4993.6	0.171	2977.5	0.036	0.014
	ENSG00000230590	1903.9	0.0	3083.3	4101.8	2739.0	5832.5	*	2739.0	0.010	0.003
	ENSG00000268001	1674.9	0.0	2847.3	2978.0	2491.4	3248.5	*	2491.4	0.019	0.002
	ENSG00000231721	1790.3	0.0	3158.0	3305.6	2356.8	4672.1	*	2356.8	0.036	0.015
	ENSG00000224078	1996.2	3.0	2941.6	3570.5	2358.9	4210.5	0.001	2355.8	0.017	0.011
	ENSG00000231074	1344.4	0.0	2587.3	2600.1	2214.4	2994.4	*	2214.4	0.014	0.004
	ENSG00000179818	1830.0	15.4	2531.5	3349.3	1992.5	3614.6	0.008	1977.1	0.007	0.013
	ENSG00000263753	1514.1	0.0	2450.2	2736.3	1820.8	3349.9	*	1820.8	0.022	0.012
	ENSG00000255717	427.2	0.0	1105.2	1887.7	1473.3	2276.2	*	1473.3	0.000	0.000
	ENSG00000225205	1083.9	0.0	2154.0	2097.5	1245.9	2743.5	*	1245.9	0.023	0.011
	ENSG00000260822	1551.8	0.0	2286.8	2605.1	1124.6	3068.3	*	1124.6	0.032	0.030
	ENSG00000226950	768.5	0.0	1695.5	2282.7	923.6	3184.5	*	923.6	0.001	0.000
	ENSG00000257599	984.8	48.5	1686.0	1867.0	664.1	2507.6	0.073	615.6	0.023	0.017
	ENSG00000206573	734.5	0.0	1933.0	2160.2	582.2	3305.9	*	582.2	0.004	0.000
	ENSG00000259834	768.0	0.0	1420.8	2051.1	455.1	3081.7	*	455.1	0.003	0.006
	ENSG00000261455	592.7	0.0	1408.8	1952.9	236.4	2662.2	*	236.4	0.001	0.002
	ENSG00000268205	694.3	0.0	1646.4	2226.1	215.9	3991.3	*	215.9	0.006	0.001
	ENSG00000257027	1156.6	0.0	2479.1	3158.2	95.8	5295.2	*	95.8	0.008	0.002
	ENSG00000245937	718.8	0.0	1599.9	1399.9	53.3	1766.0	*	53.3	0.026	0.001
	ENSG00000269893	1190.8	0.0	2532.2	2348.8	52.9	3249.9	*	52.9	0.028	0.012
	ENSG00000242086	1232.8	0.0	2457.3	2478.6	42.9	3732.6	*	42.9	0.029	0.001
	ENSG00000274020	696.3	0.0	1666.1	1622.9	20.6	2101.4	*	20.6	0.007	0.000
	ENSG00000271856	726.6	0.0	1402.5	1619.6	4.6	2052.8	*	4.6	0.005	0.004
	ENSG00000225783	694.0	0.0	1632.8	2572.5	4.1	4462.5	*	4.1	0.002	0.001
	ENSG00000215256	614.2	0.0	1339.7	1671.5	3.9	2657.8	*	3.9	0.006	0.004
	ENSG00000248242	829.7	0.0	1852.8	1949.0	2.8	2863.6	*	2.8	0.011	0.014
	ENSG00000179406	384.8	0.0	1167.8	1113.7	2.5	2344.4	*	2.5	0.029	0.020
	ENSG00000258199	513.8	0.0	1710.6	1446.7	2.1	2075.2	*	2.1	0.007	0.000
	ENSG00000223745	667.9	0.0	1437.1	1939.6	1.1	3223.7	*	1.1	0.005	0.005

ncRNA	ENSG00000252010	374.7	0.6	787.5	1474.0	503.7	3165.5	0.001	503.1	0.009	0.000
	ENSG00000200087	146.8	0.1	287.5	342.0	97.4	570.0	0.001	97.3	0.017	0.045
mRNA	ENSG00000081377	223.7	158.2	173.6	327.7	306.9	211.0	0.516	148.6	0.003	0.005
	ENSG00000122218	138.5	107.6	102.3	201.7	205.7	105.8	0.523	98.1	0.001	0.001
	ENSG00000168685	104.8	77.3	128.9	259.1	171.0	298.5	0.452	93.8	0.000	0.000
	ENSG00000073849	139.5	110.5	114.6	206.9	201.7	117.5	0.548	91.2	0.002	0.000
	ENSG00000188994	100.4	84.4	84.8	175.1	173.0	84.0	0.488	88.5	0.000	0.000
	ENSG00000114098	111.4	86.9	110.1	165.1	160.8	93.7	0.540	73.9	0.004	0.001
	ENSG00000078142	80.5	66.1	82.4	136.0	139.0	93.8	0.476	72.8	0.001	0.001
	ENSG00000198700	33.7	0.3	49.7	70.0	72.4	57.7	0.004	72.1	0.000	0.000
	ENSG00000096433	50.7	0.2	64.7	75.2	72.1	74.2	0.003	72.0	0.051	0.030
	ENSG00000100307	97.8	69.2	115.6	151.9	140.5	96.2	0.492	71.3	0.006	0.000
	ENSG00000103365	94.7	77.6	87.3	163.9	148.5	113.3	0.523	70.9	0.000	0.000
	ENSG00000109861	46.3	9.1	58.3	70.5	79.2	50.5	0.114	70.1	0.015	0.009
	ENSG00000185753	40.8	1.1	55.1	76.2	71.1	62.0	0.016	70.0	0.001	0.000
	ENSG00000167895	116.0	79.5	136.4	217.2	149.5	230.9	0.532	70.0	0.003	0.001
	ENSG00000196092	115.5	80.7	125.8	175.1	150.6	147.2	0.536	69.9	0.017	0.010
	ENSG00000180694	48.7	0.1	65.6	70.8	68.5	58.7	0.002	68.4	0.051	0.006
	ENSG00000160613	44.2	0.1	62.7	94.8	68.5	98.6	0.002	68.4	0.001	0.001
	ENSG00000172667	51.4	18.3	70.0	86.7	86.6	64.7	0.211	68.3	0.004	0.000
	ENSG00000138802	37.9	1.1	46.7	68.2	68.8	55.8	0.016	67.7	0.001	0.000
	ENSG00000013725	45.5	0.7	83.9	87.4	68.4	101.1	0.011	67.6	0.013	0.000
	ENSG00000135597	46.2	22.2	62.7	87.4	89.6	69.0	0.248	67.4	0.001	0.000
	ENSG00000114857	32.9	0.1	47.2	90.6	66.4	102.8	0.002	66.3	0.000	0.000
	ENSG00000079459	40.6	2.0	53.1	67.6	68.3	52.4	0.030	66.3	0.005	0.003
	ENSG00000111885	56.6	38.4	76.9	99.7	103.0	68.4	0.373	64.6	0.001	0.000
	ENSG00000165672	40.4	2.6	50.8	62.0	66.7	44.2	0.039	64.2	0.013	0.004
	ENSG00000134313	38.1	0.2	52.1	70.4	63.9	52.2	0.003	63.8	0.001	0.000
	ENSG00000278311	26.9	0.1	43.2	61.3	63.8	51.8	0.001	63.7	0.000	0.000
	ENSG00000139645	48.5	1.0	67.3	70.7	64.6	58.3	0.016	63.6	0.053	0.005
	ENSG00000156738	35.5	0.1	52.4	76.3	63.5	74.9	0.002	63.4	0.001	0.000
	ENSG00000109436	37.7	0.3	50.8	64.3	63.2	55.4	0.004	63.0	0.006	0.001
	ENSG00000198380	40.9	1.0	60.6	60.8	63.8	49.3	0.015	62.8	0.049	0.004
	ENSG00000062598	43.5	4.9	52.7	71.1	67.7	57.0	0.073	62.8	0.006	0.002

	ENSG00000138798	83.6	57.8	88.6	136.1	120.1	116.1	0.481	62.3	0.005	0.004
	ENSG00000125827	44.0	14.7	53.0	88.4	77.1	69.2	0.191	62.3	0.000	0.000
	ENSG00000115977	81.2	67.9	74.3	165.6	129.8	156.9	0.523	61.9	0.000	0.000
	ENSG00000183020	40.0	0.4	58.0	69.5	61.9	57.9	0.006	61.5	0.005	0.001
	ENSG00000155561	33.7	0.0	61.2	61.3	61.1	53.2	0.000	61.1	0.009	0.000
	ENSG00000101940	47.1	2.7	64.8	74.5	63.5	71.3	0.043	60.8	0.027	0.012
	ENSG00000126457	34.7	0.1	57.0	63.1	60.6	50.8	0.001	60.6	0.004	0.000
	ENSG00000197381	38.4	0.1	53.9	68.6	60.5	54.1	0.002	60.4	0.002	0.000
	ENSG00000138668	36.0	4.3	47.8	62.1	64.4	52.9	0.067	60.1	0.005	0.001
	ENSG00000173273	34.5	0.0	65.3	73.0	60.0	59.8	0.000	60.0	0.001	0.000
	ENSG00000104231	47.3	0.2	64.5	76.5	60.1	70.6	0.004	59.9	0.017	0.002
	ENSG00000141456	37.3	8.1	45.5	61.7	67.7	50.4	0.119	59.7	0.005	0.006
	ENSG00000152689	43.0	18.1	56.1	82.2	77.4	63.6	0.234	59.2	0.000	0.000
	ENSG00000182957	73.1	50.7	75.1	106.0	109.9	78.8	0.461	59.2	0.019	0.014
	ENSG00000197976	43.1	0.1	56.0	65.9	59.2	58.3	0.002	59.0	0.028	0.007
	ENSG00000275126	44.6	5.0	61.8	66.2	63.5	62.1	0.078	58.6	0.054	0.014
	ENSG00000163902	55.6	38.1	63.9	99.1	96.5	65.0	0.394	58.5	0.000	0.000
	ENSG00000156504	53.1	35.2	61.7	96.7	93.5	69.9	0.376	58.3	0.000	0.000
	ENSG00000108433	33.1	0.6	43.7	58.7	58.8	49.8	0.010	58.3	0.003	0.001
	ENSG00000143776	35.5	0.3	46.8	59.4	58.5	46.4	0.005	58.2	0.005	0.000
	ENSG00000182093	70.3	17.5	91.7	106.4	75.5	95.1	0.232	57.9	0.033	0.011
	ENSG00000110931	85.8	68.0	80.8	130.9	125.9	106.9	0.540	57.9	0.009	0.006
	ENSG00000137628	34.3	0.7	44.5	67.8	58.0	63.4	0.012	57.3	0.001	0.001
	ENSG00000196865	37.2	3.2	51.2	60.1	60.5	49.1	0.053	57.3	0.012	0.001
	ENSG00000145916	36.4	0.1	49.7	59.8	57.3	51.0	0.001	57.2	0.011	0.003
	ENSG00000170248	61.2	46.8	67.8	102.2	104.0	73.8	0.450	57.2	0.002	0.000
	ENSG00000092020	28.4	0.1	43.5	52.5	57.2	42.6	0.002	57.1	0.002	0.001
	ENSG00000141699	43.6	19.6	68.3	81.7	76.3	57.6	0.257	56.7	0.001	0.000
	ENSG00000204713	41.6	11.1	59.4	75.5	67.8	57.6	0.163	56.7	0.002	0.000
	ENSG00000071575	45.3	21.0	60.9	81.4	77.5	66.7	0.271	56.5	0.002	0.000
	ENSG00000162144	55.0	38.5	64.1	112.3	94.8	75.8	0.406	56.3	0.000	0.000
	ENSG00000112159	44.9	21.5	57.1	98.6	77.8	92.7	0.277	56.3	0.000	0.000
	ENSG00000172943	38.4	2.3	50.4	65.0	58.5	61.3	0.039	56.2	0.009	0.006
	ENSG00000183291	30.7	0.0	47.1	58.8	56.2	56.9	0.001	56.2	0.003	0.000

	ENSG00000072364	59.4	55.9	62.3	111.9	112.0	68.9	0.499	56.2	0.000	0.000
	ENSG00000066084	80.3	55.3	80.6	112.3	111.2	70.9	0.497	56.0	0.021	0.008
	ENSG00000077235	42.0	4.8	52.0	71.7	60.5	60.4	0.080	55.6	0.004	0.002
	ENSG00000160679	33.0	2.1	44.1	57.3	57.5	50.1	0.037	55.4	0.005	0.003
	ENSG00000048405	37.8	2.3	50.9	66.9	57.4	55.4	0.040	55.1	0.003	0.001
	ENSG00000090615	85.6	59.1	94.7	121.0	114.2	80.9	0.518	55.1	0.028	0.002
	ENSG00000076641	42.9	5.7	58.5	82.1	60.7	86.2	0.094	55.0	0.004	0.001
	ENSG00000079335	31.0	0.1	44.8	61.6	55.0	48.6	0.001	54.9	0.000	0.000
	ENSG00000169018	27.6	0.1	40.4	59.4	54.9	48.2	0.001	54.8	0.000	0.000
	ENSG00000129187	41.9	1.4	50.3	61.3	55.9	51.1	0.026	54.5	0.035	0.013
	ENSG00000144036	57.9	57.2	60.0	106.6	111.6	64.8	0.512	54.4	0.000	0.000
	ENSG00000175662	53.0	15.2	78.3	83.5	69.5	68.4	0.219	54.3	0.023	0.001
	ENSG00000144381	34.7	0.2	53.3	63.5	54.0	53.9	0.004	53.7	0.003	0.000
	ENSG00000126216	35.5	0.8	51.5	56.8	54.5	42.0	0.015	53.7	0.013	0.001
	ENSG00000155657	54.7	4.2	75.8	116.7	57.9	167.6	0.072	53.7	0.009	0.014
	ENSG00000015676	45.1	0.2	70.3	75.0	53.9	83.8	0.003	53.7	0.033	0.017
	ENSG00000100888	78.8	64.3	77.9	115.5	117.8	74.2	0.545	53.5	0.008	0.003
	ENSG00000102054	32.8	0.0	45.2	55.1	53.5	50.7	0.000	53.5	0.011	0.002
	ENSG00000180900	41.9	10.5	56.9	66.7	63.7	62.0	0.165	53.2	0.022	0.007
	ENSG00000054793	39.7	1.5	53.8	68.1	54.7	66.2	0.028	53.2	0.010	0.002
	ENSG00000064201	35.9	0.3	47.3	74.7	53.3	80.5	0.006	53.0	0.001	0.001
	ENSG00000152223	49.1	8.5	61.6	76.5	61.4	79.8	0.138	52.9	0.034	0.016
	ENSG00000110057	35.8	0.0	61.4	58.0	52.7	56.3	0.000	52.7	0.038	0.003
	ENSG00000255302	35.3	0.1	49.9	59.4	52.5	49.4	0.001	52.5	0.008	0.001
	ENSG00000085511	37.5	2.8	54.5	59.2	55.2	52.3	0.050	52.5	0.025	0.004
	ENSG00000157570	74.1	55.4	83.6	117.7	107.8	113.3	0.514	52.4	0.016	0.014
	ENSG00000145996	36.9	0.8	55.4	57.3	53.2	48.7	0.016	52.3	0.032	0.003
	ENSG00000149792	38.4	7.8	49.8	65.9	59.9	48.3	0.130	52.1	0.002	0.000
	ENSG00000146731	38.8	11.8	48.2	75.5	63.8	63.9	0.185	52.0	0.000	0.000
	ENSG00000182162	41.8	0.1	66.6	70.3	51.8	80.0	0.002	51.7	0.033	0.005
	ENSG00000132182	83.8	56.2	102.9	137.9	107.9	152.2	0.521	51.6	0.022	0.019
	ENSG00000084693	47.6	7.7	61.7	70.5	59.2	64.9	0.129	51.5	0.046	0.007
	ENSG00000185219	29.4	0.1	43.8	60.7	51.5	56.5	0.001	51.4	0.001	0.000
	ENSG00000109133	39.4	3.8	51.9	59.9	55.2	49.6	0.070	51.3	0.027	0.008

	ENSG00000001461	30.1	0.0	47.5	58.5	51.2	58.4	0.001	51.1	0.004	0.001
	ENSG00000108599	38.9	1.3	52.3	63.7	52.3	59.8	0.024	51.1	0.015	0.003
	ENSG00000187994	28.9	0.1	38.4	53.1	50.8	49.9	0.001	50.8	0.003	0.006
	ENSG00000136152	33.4	0.0	46.7	52.9	50.5	45.8	0.001	50.5	0.021	0.006
	ENSG00000153944	37.6	2.0	49.3	59.0	52.5	54.3	0.039	50.5	0.023	0.010
	ENSG00000180901	35.0	0.1	56.6	55.2	50.4	48.9	0.001	50.3	0.037	0.003
	ENSG00000198198	60.2	22.1	75.0	92.6	72.0	83.5	0.307	49.9	0.025	0.009
	ENSG00000130813	30.1	0.0	51.7	58.7	49.9	48.3	0.001	49.9	0.002	0.000
	ENSG00000166669	33.7	0.1	47.3	57.3	49.7	55.9	0.002	49.6	0.012	0.005
	ENSG00000154945	31.9	0.3	42.3	53.2	49.9	45.7	0.007	49.6	0.008	0.003
	ENSG00000106785	65.6	48.5	69.9	98.7	98.1	78.5	0.495	49.6	0.014	0.005
	ENSG00000147457	51.6	37.5	60.4	101.5	87.0	76.8	0.431	49.6	0.000	0.000
	ENSG00000138641	41.0	8.4	51.7	62.7	57.4	54.8	0.146	49.0	0.025	0.008
	ENSG00000113658	38.9	3.0	54.2	64.3	52.0	60.5	0.059	49.0	0.015	0.006
	ENSG00000034677	61.0	38.7	75.5	97.4	87.5	72.6	0.442	48.8	0.007	0.001
	ENSG00000134453	29.0	0.0	41.5	51.7	48.8	38.7	0.000	48.8	0.002	0.000
	ENSG00000135829	70.1	48.1	73.7	107.4	96.8	79.8	0.497	48.7	0.008	0.007
	ENSG00000156650	30.0	0.0	38.8	49.4	48.5	41.6	0.001	48.5	0.008	0.012
	ENSG00000140400	40.4	6.3	54.8	64.4	54.7	59.6	0.115	48.4	0.021	0.003
	ENSG00000147853	36.4	0.9	48.5	57.4	49.3	50.0	0.018	48.4	0.019	0.008
	ENSG00000110651	42.6	6.6	58.8	63.5	54.8	58.4	0.121	48.2	0.050	0.005
	ENSG00000134198	33.7	0.1	45.5	52.5	48.1	50.3	0.001	48.1	0.030	0.010
	ENSG00000118960	31.2	0.0	43.8	51.2	47.9	43.8	0.000	47.9	0.012	0.002
	ENSG00000074966	26.2	0.0	43.8	52.2	47.9	57.1	0.000	47.9	0.005	0.000
	ENSG00000060749	31.5	0.0	50.2	49.8	47.8	48.6	0.000	47.8	0.042	0.004
	ENSG00000141030	43.0	19.2	52.6	71.7	66.9	55.0	0.287	47.7	0.004	0.001
	ENSG00000125447	49.4	29.6	57.0	82.5	77.1	64.9	0.383	47.5	0.003	0.003
	ENSG00000120029	37.5	8.5	49.6	60.2	56.0	51.6	0.152	47.5	0.014	0.004
	ENSG00000084463	35.4	0.7	49.7	55.9	48.0	47.0	0.014	47.3	0.020	0.002
	ENSG00000133812	29.3	0.7	42.0	49.7	47.9	45.2	0.014	47.2	0.010	0.002
	ENSG00000132478	39.3	10.0	53.7	62.4	57.2	53.7	0.174	47.2	0.019	0.001
	ENSG00000141646	46.2	31.8	52.7	80.4	79.0	66.1	0.403	47.2	0.002	0.002
	ENSG00000135930	41.1	21.2	51.6	65.5	68.3	43.4	0.310	47.1	0.005	0.001
	ENSG00000100100	31.6	0.0	54.1	86.4	47.1	139.6	0.000	47.1	0.005	0.001

	ENSG00000188647	41.3	13.5	53.4	64.9	60.6	54.5	0.223	47.0	0.017	0.004
	ENSG00000125868	46.9	19.0	58.2	73.2	65.9	59.2	0.288	46.9	0.014	0.003
	ENSG00000122557	70.6	53.8	76.3	97.8	100.5	55.7	0.535	46.7	0.026	0.003
	ENSG00000157637	49.3	15.2	63.4	72.4	61.9	68.2	0.246	46.7	0.053	0.009
	ENSG00000141252	58.5	41.6	59.6	92.7	88.2	66.5	0.472	46.6	0.003	0.004
	ENSG00000263001	44.4	16.8	54.8	65.9	63.3	53.7	0.265	46.5	0.030	0.014
	ENSG00000165525	33.8	1.7	48.2	51.3	48.2	48.1	0.035	46.5	0.044	0.007
	ENSG00000107077	52.6	32.4	67.3	83.1	78.8	69.2	0.411	46.4	0.014	0.003
	ENSG00000166398	31.8	2.0	45.0	51.9	48.3	56.7	0.040	46.3	0.030	0.008
	ENSG00000105186	37.8	7.5	47.1	57.1	53.6	48.6	0.139	46.2	0.027	0.008
	ENSG00000146285	19.8	0.0	35.2	56.8	46.1	60.1	0.000	46.1	0.000	0.000
	ENSG00000242259	32.7	0.6	39.6	49.7	46.6	38.9	0.013	45.9	0.017	0.009
	ENSG00000088930	59.8	47.8	64.1	89.7	93.7	65.3	0.510	45.9	0.011	0.007
	ENSG00000111860	33.4	0.0	47.8	56.5	45.9	61.2	0.001	45.9	0.020	0.007
	ENSG00000146425	26.2	0.0	43.6	50.1	45.8	48.5	0.000	45.8	0.005	0.000
	ENSG00000102401	35.9	8.4	45.3	53.6	54.2	42.4	0.155	45.8	0.027	0.007

Supplemental Table 2. Univariate significance test to identify cofounding factors for differentially expressed genes which are affected by anti-VEGF treatment. A, C, E, G, I) Comparison between control vs nAMD. B, D, F, H, J) Comparison between baseline and anti-VEGF (sample taken when patient is on anti-VEGF treatment). Abbreviations: BMI, body mass index; blood.pressure, blood pressure medication in use; nAMD, neovascular age-related macular deneration; lncRNA, long non-coding ribonucleic acid; mRNA, messenger ribonucleic acid; ncRNA non-coding RNA; VEGF, vascular endothelial growth factor

A

	lncRNA_ENSG00000175061. Control vs. nAMD				
	SS	df	MS	F	p
(Intercept)	1.56E+09	1	1.56E+09	8.2	**0.005**
AMD	4.47E+09	1	4.47E+09	23.5	**4.0E-06**
gender	4.66E+08	1	4.66E+08	2.5	0.120
BMI	2.13E+09	1	2.13E+09	11.2	**0.001**
smoking	3.77E+08	1	3.77E+08	2.0	0.162
blood.pressure	2.76E+08	1	2.76E+08	1.5	0.231
anticholesterol	2.47E+08	1	2.47E+08	1.3	0.257
anticoagulants	5.53E+08	1	5.53E+08	2.9	0.091
antiaggregation	2.19E+08	1	2.19E+08	1.2	0.285
Residuals	2.17E+10	114	1.90E+08		

B

	lncRNA_ENSG00000175061. Baseline vs. anti-VEGF				
	SS	df	MS	F	p
(Intercept)	2.728E+08	1	2.73E+08	3.4	**0.067**
VEGF	5.905E+08	1	5.90E+08	7.4	**0.008**
gender	5.378E+08	1	5.38E+08	6.7	**0.011**
BMI	2.644E+08	1	2.64E+08	3.3	0.071
smoking	2.389E+08	1	2.39E+08	3.0	0.086
blood.pressure	1.844E+08	1	1.84E+08	2.3	0.131
anticholesterol	2.053E+08	1	2.05E+08	2.6	0.112
anticoagulants	2.974E+08	1	2.97E+08	3.7	0.056
antiaggregation	1.565E+08	1	1.56E+08	2.0	0.164
Residuals	9.103E+09	114	7.99E+07		

C

	mRNA_ENSG00000141293. Control vs. nAMD				
	SS	df	MS	F	p
(Intercept)	11058.5	1	11058.5	4.4	**0.037**
AMD	12170.6	1	12170.6	4.9	**0.029**
gender	2568.3	1	2568.3	1.0	0.312
BMI	3797.7	1	3797.7	1.5	0.220
smoking	3080.1	1	3080.1	1.2	0.269
blood.pressure	3396.2	1	3396.2	1.4	0.246
anticholesterol	3265.2	1	3265.2	1.3	0.255
anticoagulants	20551.0	1	20551.0	8.2	**0.005**
antiaggregation	8079.3	1	8079.3	3.2	**0.075**
Residuals	284460.5	114	2495.3		

D

	mRNA_ENSG00000141293. Baseline vs. anti-VEGF				
	SS	df	MS	F	p
(Intercept)	2951.1	1	2951.1	6.5	**0.012**
VEGF	10902.1	1	10902.1	23.8	**3.4E-06**
gender	977.5	1	977.5	2.1	0.146
BMI	965.7	1	965.7	2.1	0.149
smoking	1460.9	1	1460.9	3.2	0.077
blood.pressure	1101.6	1	1101.6	2.4	0.123
anticholesterol	3523.4	1	3523.4	7.7	**0.006**
anticoagulants	4937.3	1	4937.3	10.8	**0.001**
antiaggregation	3451.1	1	3451.1	7.5	**0.007**
Residuals	52120.7	114	457.2		

E

	mRNA_ENSG00000076641. Control vs. nAMD				
	SS	df	MS	F	p
(Intercept)	21526.4	1	21526.4	4.6	**0.035**
AMD	42511.7	1	42511.7	9.0	**0.003**
gender	7592.6	1	7592.6	1.6	0.208
BMI	27788.1	1	27788.1	5.9	0.017
smoking	11922.8	1	11922.8	2.5	0.115
blood.pressure	5228.6	1	5228.6	1.1	0.295
anticholesterol	8091.5	1	8091.5	1.7	0.193
anticoagulants	24094.7	1	24094.7	5.1	**0.026**
antiaggregation	13136.9	1	13136.9	2.8	0.098
Residuals	539021.5	114	4728.3		

F

	mRNA_ENSG00000076641. Baseline vs. anti-VEGF				
	SS	df	MS	F	p
(Intercept)	5566.0	1	5566.0	4.4	**0.039**
VEGF	7594.2	1	7594.2	5.9	**0.016**
gender	3818.5	1	3818.5	3.0	0.087
BMI	4375.6	1	4375.6	3.4	0.067
smoking	4923.6	1	4923.6	3.8	**0.052**
blood.pressure	3715.7	1	3715.7	2.9	0.091
anticholesterol	3618.7	1	3618.7	2.8	0.095
anticoagulants	11623.5	1	11623.5	9.1	0.003
antiaggregation	3601.0	1	3601.0	2.8	0.096
Residuals	145823.6	114	1279.2		

G

	mRNA_ENSG00000132463. Control vs. nAMD				
	SS	df	MS	F	p
(Intercept)	20125.5	1	20125.5	7.0	**0.009**
AMD	19974.4	1	19974.4	6.9	**0.010**
gender	2557.3	1	2557.3	0.9	0.349
BMI	16678.3	1	16678.3	5.8	**0.018**
smoking	3361.2	1	3361.2	1.2	0.283
blood.pressure	11563.7	1	11563.7	4.0	**0.048**
anticholesterol	3587.8	1	3587.8	1.2	0.267
anticoagulants	3266.5	1	3266.5	1.1	0.290
antiaggregation	4012.5	1	4012.5	1.4	0.241
Residuals	328985.1	114	2885.8		

H

	mRNA_ENSG00000132463. Baseline vs. anti-VEGF				
	SS	df	MS	F	p
(Intercept)	9052.0	1	9052.0	8.1	**0.005**
VEGF	15061.9	1	15061.9	13.5	**0.000**
gender	2730.1	1	2730.1	2.4	0.120
BMI	3251.8	1	3251.8	2.9	0.090
smoking	2695.5	1	2695.5	2.4	0.123
blood.pressure	6095.5	1	6095.5	5.5	**0.021**
anticholesterol	4994.2	1	4994.2	4.5	**0.036**
anticoagulants	3475.7	1	3475.7	3.1	0.080
antiaggregation	3233.5	1	3233.5	2.9	0.091
Residuals	127066.2	114	1114.6		

I

	mRNA_ENSG00000136861. Control vs. nAMD				
	SS	df	MS	F	p
(Intercept)	20494.5	1	20494.5	9.3	**0.003**
AMD	12225.8	1	12225.8	5.6	**0.020**
gender	2200.6	1	2200.6	1.0	0.319
BMI	2577.0	1	2577.0	1.2	0.281
smoking	1625.9	1	1625.9	0.7	0.391
blood.pressure	2089.3	1	2089.3	1.0	0.332
anticholesterol	6840.2	1	6840.2	3.1	0.080
anticoagulants	3723.5	1	3723.5	1.7	0.196
antiaggregation	8024.3	1	8024.3	3.7	0.058
Residuals	250422.6	114	2196.7		

J

	mRNA_ENSG00000136861. Baseline vs. anti-VEGF				
	SS	df	MS	F	p
(Intercept)	2842.2	1	2842.2	4.1	**0.046**
VEGF	11127.3	1	11127.3	15.9	**1.2E-04**
gender	1229.1	1	1229.1	1.8	0.187
BMI	3610.4	1	3610.4	5.2	**0.025**
smoking	1416.1	1	1416.1	2.0	0.157
blood.pressure	1202.9	1	1202.9	1.7	0.192
anticholesterol	3679.0	1	3679.0	5.3	**0.024**
anticoagulants	6799.3	1	6799.3	9.7	**0.002**
antiaggregation	3053.4	1	3053.4	4.4	**0.039**
Residuals	79583.4	114	698.1		

Supplemental table S3. Univarite significance test to identify cofounding factors for differentially expressed genes in atrophy analysis. A-E) Comparison between control vs nAMD F-J) Comparison between different atrophy levels. Only those univarite significance test sets are shown, which have significant values in at least one of the following paramenters: gender, BMI, smoking, blood pressure medication, anticholesterol medication, anticoagulation medication, antiaggregation medication. Abbreviations: BMI, body mass index; blood.pressure, blood pressure medication in use; nAMD, neovascular age-related macular deneration; lncRNA, long non-coding ribonucleic acid; mRNA, messenger ribonucleic acid; ncRNA non-coding RNA

A

	mRNA7_ENSG00000111266. Control vs. nAMD				
	SS	df	MS	F	p
(Intercept)	1229.9	1	1229.9	0.7	0.409
AMD	13725.3	1	13725.3	7.7	**0.007**
gender	3441.4	1	3441.4	1.9	0.168
BMI	8258.8	1	8258.8	4.6	**0.034**
smoking	6140.3	1	6140.3	3.4	0.066
blood.pressure	2365.0	1	2365.0	1.3	0.253
anticholesterol	1927.2	1	1927.2	1.1	0.301
anticoagulants	1502.9	1	1502.9	0.8	0.361
antiaggregation	5936.4	1	5936.4	3.3	0.071
Residuals	203890.2	114	1788.5		

B

	mRNA8_ENSG00000164654. Control vs. nAMD				
	SS	df	MS	F	p
(Intercept)	3097.1	1	3097.1	3.9	**0.052**
AMD	5973.4	1	5973.4	7.4	**0.007**
gender	1107.4	1	1107.4	1.4	0.243
BMI	3514.6	1	3514.6	4.4	**0.039**
smoking	1344.1	1	1344.1	1.7	0.198
blood.pressure	1608.3	1	1608.3	2.0	0.160
anticholesterol	908.9	1	908.9	1.1	0.290
anticoagulants	1071.4	1	1071.4	1.3	0.250
antiaggregation	885.7	1	885.7	1.1	0.296
Residuals	91541.2	114	803.0		

C

	mRNA9_ENSG00000106344. Control vs. nAMD				
	SS	df	MS	F	p
(Intercept)	4251.2	1	4251.2	3.2	0.079
AMD	9044.0	1	9044.0	6.7	**0.011**
gender	1129.4	1	1129.4	0.8	0.362
BMI	6989.2	1	6989.2	5.2	**0.025**
smoking	1831.8	1	1831.8	1.4	0.246
blood.pressure	3039.2	1	3039.2	2.3	0.136
anticholesterol	3788.3	1	3788.3	2.8	0.097
anticoagulants	1835.2	1	1835.2	1.4	0.246
antiaggregation	3257.5	1	3257.5	2.4	0.123
Residuals	153778.5	114	1348.9		

D

	mRNA10_ENSG00000100023. Control vs. nAMD				
	SS	df	MS	F	p
(Intercept)	34903.0	1	34903.0	17.4	**5.9E-05**
AMD	18323.2	1	18323.2	9.1	**0.003**
gender	10922.9	1	10922.9	5.4	**0.021**
BMI	11735.6	1	11735.6	5.9	**0.017**
smoking	3398.0	1	3398.0	1.7	0.196
blood.pressure	12268.7	1	12268.7	6.1	**0.015**
anticholesterol	2545.4	1	2545.4	1.3	0.262
anticoagulants	8360.7	1	8360.7	4.2	**0.043**
antiaggregation	2447.9	1	2447.9	1.2	0.272
Residuals	228581.7	114	2005.1		

E

	mRNA12_ENSG00000102606. Control vs. nAMD				
	SS	df	MS	F	p
(Intercept)	9668.1	1	9668.1	3.2	0.077
AMD	16499.9	1	16499.9	5.4	**0.022**
gender	5360.0	1	5360.0	1.8	0.187
BMI	4158.5	1	4158.5	1.4	0.245
smoking	13404.4	1	13404.4	4.4	**0.038**
blood.pressure	3694.2	1	3694.2	1.2	0.273
anticholesterol	3433.0	1	3433.0	1.1	0.291
anticoagulants	11335.9	1	11335.9	3.7	0.056
antiaggregation	9034.6	1	9034.6	3.0	0.088
Residuals	347168.2	114	3045.3		

F

	mRNA1_ENSG00000137200. Atrophy				
	SS	df	MS	F	p
(Intercept)	1926.5	1	1926.5	1.8	0.181
atrophy	14134.5	4	3533.6	3.4	**0.017**
gender	5646.5	1	5646.5	5.4	**0.025**
BMI	1906.5	1	1906.5	1.8	0.183
smoking	6605.0	1	6605.0	6.3	**0.016**
blood.pressure	910.6	1	910.6	0.9	0.355
anticholesterol	1681.2	1	1681.2	1.6	0.211
anticoagulants	1333.9	1	1333.9	1.3	0.264
antiaggregation	3726.7	1	3726.7	3.6	0.065
Residuals	46978.9	45	1043.975		

G

	mRNA2_ENSG00000178562. Atrophy				
	SS	df	MS	F	p
(Intercept)	3539.9	1	3539.9	3.1	0.087
atrophy	16393.0	4	4098.2	3.5	**0.013**
gender	5103.1	1	5103.1	4.4	**0.041**
BMI	1566.8	1	1566.8	1.4	0.250
smoking	1097.6	1	1097.6	0.9	0.335
blood.pressure	1881.5	1	1881.5	1.6	0.208
anticholesterol	678.3	1	678.3	0.6	0.448
anticoagulants	4726.2	1	4726.2	4.1	**0.049**
antiaggregation	1747.6	1	1747.6	1.5	0.225
Residuals	51996.4	45	1155.5		

H

	mRNA3_ENSG00000242372. Atrophy				
	SS	df	MS	F	p
(Intercept)	5194.4	1	5194.4	4.9	**0.032**
atrophy	15559.5	4	3889.9	3.7	**0.011**
gender	2151.6	1	2151.6	2.0	0.161
BMI	7876.8	1	7876.8	7.4	**0.009**
smoking	6287.8	1	6287.8	5.9	**0.019**
blood.pressure	1454.7	1	1454.7	1.4	0.248
anticholesterol	1506.9	1	1506.9	1.4	0.239
anticoagulants	2201.3	1	2201.3	2.1	0.156
antiaggregation	1496.0	1	1496.0	1.4	0.241
Residuals	47695.7	45	1059.9		

I

	mRNA8_ENSG00000164654. Atrophy				
	SS	df	MS	F	p
(Intercept)	3834.0	1	3834.0	8.3	**0.006**
atrophy	7172.9	4	1793.2	3.9	**0.009**
gender	1166.5	1	1166.5	2.5	0.120
BMI	632.4	1	632.4	1.4	0.249
smoking	892.5	1	892.5	1.9	0.172
blood.pressure	2081.3	1	2081.3	4.5	**0.040**
anticholesterol	1913.5	1	1913.5	4.1	**0.048**
anticoagulants	690.1	1	690.1	1.5	0.229
antiaggregation	524.6	1	524.6	1.1	0.293
Residuals	20887.6	45	464.2		

J

	mRNA11_ENSG00000173674. Atrophy				
	SS	df	MS	F	p
(Intercept)	4377.3	1	4377.3	2.4	0.127
atrophy	23703.2	4	5925.8	3.3	**0.019**
gender	7410.4	1	7410.4	4.1	**0.049**
BMI	4322.1	1	4322.1	2.4	0.129
smoking	3882.2	1	3882.2	2.1	0.150
blood.pressure	6080.0	1	6080.0	3.4	0.073
anticholesterol	18237.1	1	18237.1	10.1	**0.003**
anticoagulants	2265.9	1	2265.9	1.3	0.269
antiaggregation	3212.1	1	3212.1	1.8	0.190
Residuals	81450.3	45	1810.0		

Supplemental table S4. Univarite significance test to identify cofounding factors for differentially expressed genes in fluids analysis. A-G) Comparison between control vs nAMD H-P) Comparison between SRF and IRF. Only those univarite significance test sets are shown, which have significant values in at least one of the following paramenters: gender, BMI, smoking, blood pressure medication, anticholesterol medication, anticoagulation medication, antiaggregation medication. Abbreviations: SRF, subretinal fluid; IRF, intraretinal fluid; BMI, body mass index, blood.pressure, blood pressure medication in use; nAMD, neovascular age-related macular deneration; lncRNA, long non-coding ribonucleic acid; mRNA, messenger ribonucleic acid; ncRNA non-coding RNA

A

	lncRNA2_ENSG00000177406. Control vs. nAMD				
	SS	df	MS	F	p
(Intercept)	32706582.7	1	32706582.7	4.5	**0.036**
AMD	24015654.1	1	24015654.1	3.3	0.071
gender	9130022.63	1	9130022.6	1.3	0.264
BMI	35046625.5	1	35046625.5	4.8	**0.030**
smoking	15059182.2	1	15059182.2	2.1	0.152
blood.pressure	9633858.99	1	9633859.0	1.3	0.251
anticholesterol	22185276.9	1	22185276.9	3.1	0.083
anticoagulants	4879649.69	1	4879649.7	0.7	0.413
antiaggregation	28781793.8	1	28781793.8	4.0	**0.049**
Residuals	825599447	114	7242100.4		

B

	mRNA1_ENSG00000241258. Control vs nAMD				
	SS	df	MS	F	p
(Intercept)	23641.9	1	23641.9	14.6	**2.2E-04**
AMD	3254.0	1	3254.0	2.0	0.159
gender	3285.8	1	3285.8	2.0	0.157
BMI	1339.1	1	1339.1	0.8	0.365
smoking	2628.8	1	2628.8	1.6	0.205
blood.pressure	9614.2	1	9614.2	5.9	**0.016**
anticholesterol	1689.7	1	1689.7	1.0	0.309
anticoagulants	2044.9	1	2044.9	1.3	0.263
antiaggregation	3247.1	1	3247.1	2.0	0.159
Residuals	184608.1	114	1619.4		

C

	mRNA3_ENSG00000102901. Control vs. nAMD				
	SS	df	MS	F	p
(Intercept)	10949.8	1	10949.8	2.5	0.113
AMD	28627.9	1	28627.9	6.7	**0.011**
gender	4834.9	1	4834.9	1.1	0.291
BMI	7570.8	1	7570.8	1.8	0.187
smoking	14048.1	1	14048.1	3.3	0.073
blood.pressure	7934.9	1	7934.9	1.8	0.177
anticholesterol	17671.7	1	17671.7	4.1	**0.045**
anticoagulants	4776.8	1	4776.8	1.1	0.294
antiaggregation	6735.1	1	6735.1	1.6	0.213
Residuals	490248.4	114	4300.4		

D

	mRNA4_ENSG00000198959. Control vs. nAMD				
	SS	df	MS	F	p
(Intercept)	23825.9	1	23825.9	7.6	**0.007**
AMD	14718.7	1	14718.7	4.7	**0.032**
gender	2599.4	1	2599.4	0.8	0.365
BMI	3364.1	1	3364.1	1.1	0.303
smoking	4570.4	1	4570.4	1.5	0.230
blood.pressure	2502.9	1	2502.9	0.8	0.374
anticholesterol	13788.6	1	13788.6	4.4	**0.038**
anticoagulants	3574.8	1	3574.8	1.1	0.288
antiaggregation	3291.8	1	3291.8	1.0	0.308
Residuals	357907.7	114	3139.5		

E

	mRNA6_ENSG00000125827. Control vs. nAMD				
	SS	df	MS	F	p
(Intercept)	40600.9	1	40600.9	11.6	**0.001**
AMD	56187.2	1	56187.2	16.0	**1.1E-04**
gender	19130.8	1	19130.8	5.4	**0.021**
BMI	2726.3	1	2726.3	0.8	0.380
smoking	4807.8	1	4807.8	1.4	0.245
blood.pressure	12115.7	1	12115.7	3.4	0.066
anticholesterol	3197.1	1	3197.1	0.9	0.342
anticoagulants	5936.4	1	5936.4	1.7	0.196
antiaggregation	7810.1	1	7810.1	2.2	0.139
Residuals	400616.0	114	3514.2		

F

	mRNA8_ENSG00000176108. Control vs. nAMD				
	SS	df	MS	F	p
(Intercept)	28106.9	1	28106.9	7.9	**0.006**
AMD	7796.0	1	7796.0	2.2	0.142
gender	5665.4	1	5665.4	1.6	0.210
BMI	7887.8	1	7887.8	2.2	0.140
smoking	3716.7	1	3716.7	1.0	0.310
blood.pressure	4884.7	1	4884.7	1.4	0.244
anticholesterol	16581.5	1	16581.5	4.6	**0.033**
anticoagulants	5292.0	1	5292.0	1.5	0.226
antiaggregation	2967.0	1	2967.0	0.8	0.364
Residuals	406792.4	114	3568.4		

G

	mRNA20_ENSG00000182957. Control vs. nAMD				
	SS	df	MS	F	p
(Intercept)	44737.1	1	44737.1	8.2	**0.005**
AMD	29970.1	1	29970.1	5.5	**0.020**
gender	13266.1	1	13266.1	2.4	0.121
BMI	11402.8	1	11402.8	2.1	0.150
smoking	5976.3	1	5976.3	1.1	0.296
blood.pressure	12917.9	1	12917.9	2.4	0.126
anticholesterol	6789.3	1	6789.3	1.3	0.266
anticoagulants	7537.8	1	7537.8	1.4	0.241
antiaggregation	21620.0	1	21620.0	4.0	**0.048**
Residuals	618333.3	114	5424.0		

H

	mRNA1_ENSG00000241258. SRF vs. IRF				
	SS	df	MS	F	p
(Intercept)	1148.2	1	1148.2	1.5	0.232
fluid status	8397.9	2	4198.9	5.4	**0.008**
gender	1543.1	1	1543.1	2.0	0.167
BMI	835.8	1	835.8	1.1	0.307
smoking	5446.4	1	5446.4	6.9	**0.011**
blood.pressure	958.6	1	958.6	1.2	0.274
anticholesterol	2770.7	1	2770.7	3.5	0.066
anticoagulants	1216.5	1	1216.5	1.6	0.219
antiaggregation	2355.0	1	2355.0	3.0	0.090
Residuals	36051.0	46	783.7		

I

	mRNA5_ENSG00000154188. SRF vs. IRF				
	SS	df	MS	F	p
(Intercept)	968.2	1	968.2	0.6	0.426
fluid status	10645.3	2	5322.7	3.5	**0.037**
gender	1305.8	1	1305.8	0.9	0.356
BMI	3899.5	1	3899.5	2.6	0.114
smoking	1263.7	1	1263.7	0.8	0.364
blood.pressure	6200.2	1	6200.2	4.1	**0.048**
anticholesterol	3857.9	1	3857.9	2.6	0.116
anticoagulants	2271.3	1	2271.3	1.5	0.225
antiaggregation	4124.5	1	4124.5	2.7	0.104
Residuals	69092.7	46	1502.0		

J

	mRNA6_ENSG00000125827. SRF vs. IRF				
	SS	df	MS	F	p
(Intercept)	2139.2	1	2139.2	1.2	0.286
fluid status	16672.3	2	8336.1	4.5	**0.016**
gender	4791.2	1	4791.2	2.6	0.113
BMI	2753.5	1	2753.5	1.5	0.227
smoking	9543.4	1	9543.4	5.2	**0.027**
blood.pressure	10558.4	1	10558.4	5.7	**0.021**
anticholesterol	2392.6	1	2392.6	1.3	0.260
anticoagulants	5179.2	1	5179.2	2.8	0.100
antiaggregation	10059.6	1	10059.6	5.5	**0.024**
Residuals	84510.9	46	1837.2		

K

	mRNA11_ENSG00000131748. SRF vs. IRF				
	SS	df	MS	F	p
(Intercept)	2766.6	1	2766.6	2.0	0.167
fluid status	8794.9	2	4397.4	3.1	**0.053**
gender	8034.2	1	8034.2	5.7	**0.021**
BMI	3967.6	1	3967.6	2.8	0.099
smoking	2006.6	1	2006.6	1.4	0.238
blood.pressure	3626.1	1	3626.1	2.6	0.115
anticholesterol	1423.0	1	1423.0	1.0	0.319
anticoagulants	2254.2	1	2254.2	1.6	0.211
antiaggregation	4458.5	1	4458.5	3.2	0.081
Residuals	64537.5	46	1403.0		

L

	mRNA13_ENSG00000138078. SRF vs. IRF				
	SS	df	MS	F	p
(Intercept)	9229.8	1	9229.8	7.3	**0.010**
fluid status	9516.3	2	4758.2	3.8	**0.031**
gender	4254.7	1	4254.7	3.4	0.073
BMI	2285.7	1	2285.7	1.8	0.186
smoking	7852.6	1	7852.6	6.2	**0.016**
blood.pressure	1235.9	1	1235.9	1.0	0.328
anticholesterol	1680.1	1	1680.1	1.3	0.255
anticoagulants	1985.0	1	1985.0	1.6	0.217
antiaggregation	1517.5	1	1517.5	1.2	0.279
Residuals	58249.4	46	1266.3		

M

	mRNA14_ENSG00000157625. SRF vs. IRF				
	SS	df	MS	F	p
(Intercept)	1695.4	1	1695.4	1.2	0.283
fluid status	16593.1	2	8296.5	5.8	**0.006**
gender	1526.0	1	1526.0	1.1	0.308
BMI	1797.3	1	1797.3	1.3	0.269
smoking	7175.2	1	7175.2	5.0	**0.030**
blood.pressure	1852.9	1	1852.9	1.3	0.262
anticholesterol	1613.5	1	1613.5	1.1	0.295
anticoagulants	3686.6	1	3686.6	2.6	0.116
antiaggregation	1705.7	1	1705.7	1.2	0.281
Residuals	66068.3	46	1436.3		

N

	mRNA16_ENSG00000143776. SRF vs. IRF				
	SS	df	MS	F	p
(Intercept)	1695.8	1	1695.8	1.0	0.320
fluid status	13978.4	2	6989.2	4.2	**0.022**
gender	2454.2	1	2454.2	1.5	0.233
BMI	4638.9	1	4638.9	2.8	0.103
smoking	1933.3	1	1933.3	1.2	0.289
blood.pressure	3322.6	1	3322.6	2.0	0.166
anticholesterol	2665.0	1	2665.0	1.6	0.214
anticoagulants	9467.2	1	9467.2	5.6	**0.022**
antiaggregation	1993.3	1	1993.3	1.2	0.282
Residuals	77217.9	46	1678.6		

O

mRNA17_ENSG00000102401. SRF vs. IRF					
	SS	df	MS	F	p
(Intercept)	10457.7	1	10457.7	7.4	**0.009**
fluid status	11570.4	2	5785.2	4.1	**0.024**
gender	15966.7	1	15966.7	11.2	**0.002**
BMI	6472.3	1	6472.3	4.6	**0.038**
smoking	2864.8	1	2864.8	2.0	0.162
blood.pressure	1914.2	1	1914.2	1.3	0.252
anticholesterol	1933.5	1	1933.5	1.4	0.249
anticoagulants	3033.8	1	3033.8	2.1	0.151
antiaggregation	2916.9	1	2916.9	2.1	0.159
Residuals	65346.1	46	1420.6		

P

mRNA19_ENSG00000185278. SRF vs. IRF					
	SS	df	MS	F	p
(Intercept)	1131.529	1	1131.529	0.779486	0.381889
fluid status	11009.26	2	5504.63	3.79202	**0.029896**
gender	1926.755	1	1926.755	1.3273	0.255239
BMI	8070.154	1	8070.154	5.559354	**0.02269**
smoking	3086.445	1	3086.445	2.126185	0.151595
blood.pressure	2833.643	1	2833.643	1.952035	0.169071
anticholesterol	3483.868	1	3483.868	2.399961	0.128192
anticoagulants	3625.544	1	3625.544	2.497558	0.120875
antiaggregation	2948.95	1	2948.95	2.031468	0.160822
Residuals	66775.22	46	1451.635		

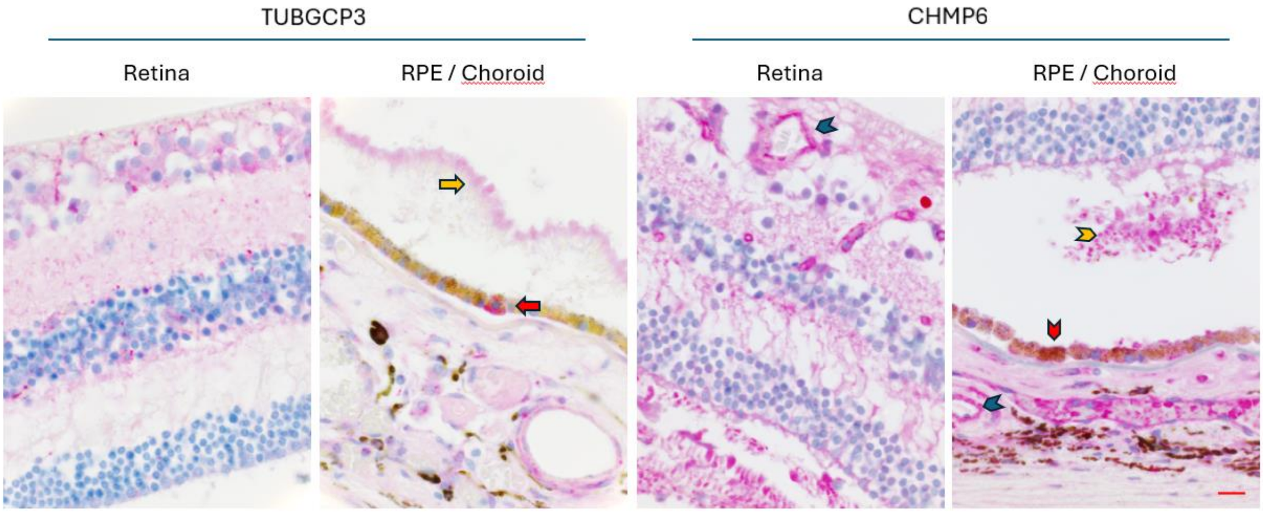

Supplemental figure S3. Staining of TUBGCP3 and CHMP6 in the human retina, RPE and choroid from cadaver samples. Arrows show staining of TUBGCP3 in photoreceptor outer segments (yellow arrow) and retinal pigment epithelial cells (red arrow). Arrowheads show staining of CHMP6 in photoreceptor outer segments (yellow arrowhead), retinal pigment epithelial cells (red arrowhead) and vascular wall (blue arrowhead). Scalebar 20 µm.
